# Fermentation stage-dependent adaptations of *Bacillus licheniformis* during enzyme production

**DOI:** 10.1186/1475-2859-12-120

**Published:** 2013-12-06

**Authors:** Sandra Wiegand, Birgit Voigt, Dirk Albrecht, Johannes Bongaerts, Stefan Evers, Michael Hecker, Rolf Daniel, Heiko Liesegang

**Affiliations:** 1 Department of Genomic and Applied Microbiology & Göttingen Genomics Laboratory, Institut für Mikrobiologie und Genetik, Norddeutsches Zentrum für Mikrobielle Genomforschung, Georg-August-Universität Göttingen, Grisebachstr. 8, D-37077 Göttingen, Germany; 2 Division of Microbial Physiology and Molecular Biology, Institut für Mikrobiologie, Norddeutsches Zentrum für Mikrobielle Genomforschung, Ernst-Moritz-Arndt-Universität Greifswald, F.-L.-Jahnstr. 15, D-17487 Greifswald, Germany; 3 Henkel AG & Co. KGaA, Henkelstr. 67, D-40191 Düsseldorf, Germany

**Keywords:** Differential gene expression, Transcriptomics, Proteomics, RNA-Seq, Subtilisin Carlsberg, Industrial production, Stress response, Sporulation, Lichenicidin

## Abstract

**Background:**

Industrial fermentations can generally be described as dynamic biotransformation processes in which microorganisms convert energy rich substrates into a desired product. The knowledge of active physiological pathways, reflected by corresponding gene activities, allows the identification of beneficial or disadvantageous performances of the microbial host. Whole transcriptome RNA-Seq is a powerful tool to accomplish in-depth quantification of these gene activities, since the low background noise and the absence of an upper limit of quantification allow the detection of transcripts with high dynamic ranges. Such data enable the identification of potential bottlenecks and futile energetic cycles, which in turn can lead to targets for rational approaches to productivity improvement. Here we present an overview of the dynamics of gene activity during an industrial-oriented fermentation process with *Bacillus licheniformis*, an important industrial enzyme producer. Thereby, valuable insights which help to understand the complex interactions during such processes are provided.

**Results:**

Whole transcriptome RNA-Seq has been performed to study the gene expression at five selected growth stages of an industrial-oriented protease production process employing a germination deficient derivative of *B. licheniformis* DSM13. Since a significant amount of genes in *Bacillus* strains are regulated posttranscriptionally, the generated data have been confirmed by 2D gel-based proteomics. Regulatory events affecting the coordinated activity of hundreds of genes have been analyzed. The data enabled the identification of genes involved in the adaptations to changing environmental conditions during the fermentation process. A special focus of the analyses was on genes contributing to central carbon metabolism, amino acid transport and metabolism, starvation and stress responses and protein secretion. Genes contributing to lantibiotics production and Tat-dependent protein secretion have been pointed out as potential optimization targets.

**Conclusions:**

The presented data give unprecedented insights into the complex adaptations of bacterial production strains to the changing physiological demands during an industrial-oriented fermentation. These are, to our knowledge, the first publicly available data that document quantifiable transcriptional responses of the commonly employed production strain *B. licheniformis* to changing conditions over the course of a typical fermentation process in such extensive depth.

## Background

For several decades, strains of the *Bacillus subtilis* group [1] have been exploited for industrial purposes. The scope of applications includes the production of amylases, proteases and antibiotics by strains of *B. subtilis*, *B. amyloliquefaciens*, *B. pumilus* or *B. licheniformis*[2]. High capacities of product secretion, high growth rates, and the GRAS (generally regarded as safe) status of many strains have contributed to the employment of these species as biotechnological workhorses [2]. In general, the production process can be considered as an energy consuming biotransformation in which a nutrient rich substrate is converted into the desired product by a member of the genus *Bacillus*.

The productive process examined in this study is based on the production platform *B. licheniformis*, which has been proven to perform well for the production of alkaline proteases and in particular subtilisins, which are used in all types of laundry detergents [3]. Therefore, research efforts have been focused on the *B. licheniformis* subtilisin fermentation process and the resulting yield of active enzyme. A major aspect has been monitoring and improvement of bioprocess parameters such as oxygen transfer rate [4-6], pH value [7,8], inoculum quality [9] and initial glucose concentration [10], whereas other studies addressed the optimization of the fermentation medium [11,12]. Strategies for the molecular biological improvement of subtilisin [13] and its secretion [14] have been described. Attention has also been paid to strain optimization by generation of deletion mutants targeting transfer of genetic material [15,16], secretion capability [17], sporulation and biological containment [18-20]. Investigation of *B. licheniformis* under different stress conditions by proteomics and microarray-based transcriptomics have been applied to identify marker genes [21-24], to enable the detection of stressors during a productive fermentation process. However, rational strain or bioprocess optimization requires potential targets and therefore the knowledge of genomic activities during the crucial stages of a fermentation process under industry-oriented conditions is essential.

An RNA-Seq-based study targeting the identification of *B. licheniformis* DSM13 RNA-based regulatory elements such as non-coding and antisense RNAs under production-oriented growth conditions has recently been published by our group [25]. The application of RNA-Seq allows the quantification of transcripts with a hitherto unmatched dynamic range spanning several orders of magnitude [26], therefore enabling in depth analysis of differential expression between physiological conditions or developmental states. Further advantages of RNA-Seq are the low background noise, the provided single base resolution and the high reproducibility [26,27]. Therefore, RNA-Seq, especially when coupled with other “omics” techniques like 2D gel-based proteomics, provides the opportunity for global investigation of microbial gene expression. However, although recent advantages in RNA-Seq technology have greatly enhanced the efficiency and availability of this approach, no such data on industrial fermentations of *B. licheniformis* have been made publicly available to this day.

To identify gene activities of *B. licheniformis* directly related to the productivity of a subtilisin fermentation process, we present a high-resolution quantitative and dynamic exploration of the transcriptional responses of *B. licheniformis* confirmed by proteome data. Special attention was given to production stage-related adaptions of *B. licheniformis*. The RNA abundances and the cytoplasmic proteome composition of all samples were determined by RNA-Seq experiments and by 2D gel electrophoresis [25], respectively. As measure of gene expression, the normalized amount of sequenced nucleotides per gene is expressed in single-base resolution by the NPKM (**n**ucleotide activity **p**er **k**ilobase of exon model per **m**illion mapped reads) value [25], which is closely related to the more common RPKM value [28]. These data provide a first analytical framework to gain better understanding of the dynamics during such fermentations, and to enable the identification of potential physiological and genetic bottlenecks. Furthermore, the data are intended as a reference for subsequent comparisons with transcriptome data from other fermentation procedures employing related *Bacillus* strains, in order to guide rational approaches for the optimization of production processes.

## Results and discussion

**Figure 1:**
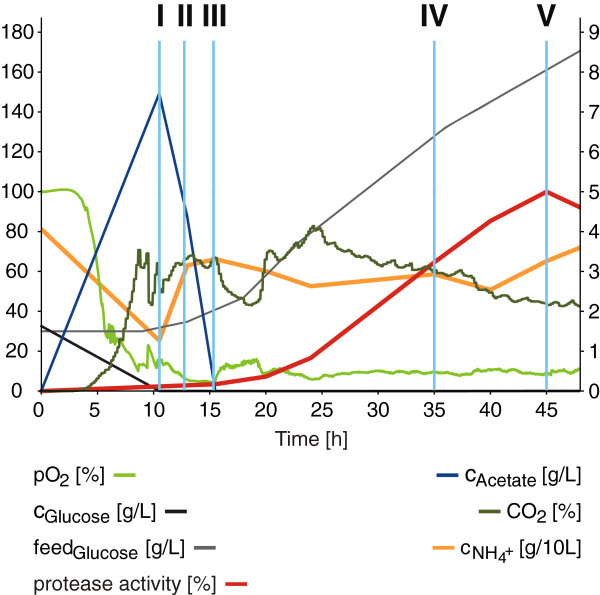
**Protease production and process parameters.** Process parameters are shown for fermentation L (please refer to Additional file 1: Figure S1 for replicate fermentations R and M). Oxygen partial pressure pO_2_ [%], glucose concentration c_Glucose_ [g/L], supplied glucose feed_Glucose_ [g/L] and normalized protease activity [%] are displayed on the left y-axis, whereas acetate concentration c_Acetate_ [g/L], carbon dioxide content CO_2_ [%], and ammonium concentration c_NH4+_ [g/10 L] are scaled on the right y-axis. The process time t [h] is given on the x-axis. The sampling points I to V are indicated by light blue lines. The figure was modified from Wiegand et al. [25] where a detailed analysis of RNA-Seq data of the same experimental setup aiming at the identification of regulatory RNAs was performed.

In this study, transcriptome and proteome data of selected samples from an industry-oriented fermentation have been analyzed with focus on physiological changes during the process. The samples were taken in triplicate at five time points (sampling points I-V) during growth within a subtilisin fermentation process of *B. licheniformis* MW3Δspo (Figure 1; Additional file 1: Figure S1). This strain is a germination deficient mutant of *B. licheniformis* DSM13, transformed with an expression plasmid encoding a subtilisin protease. Sampling point I represents the growth in presence of glucose, whereas sampling points II and III correspond to the subsequent phase of glucose starvation. Sampling points IV and V represent the productive stages of the process in which the alkaline protease is synthesized and secreted.

**Figure 2:**
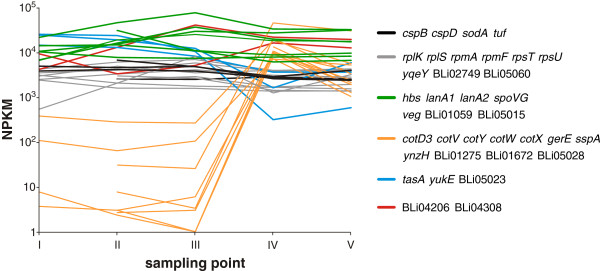
**Most abundant gene transcripts.** Mean NPKM values of the most abundant gene transcripts are plotted against sampling points. Colored lines indicate classes of similarly abundant transcripts. Grey and black lines represent genes with high RNA abundances in all 15 samples (NPKM values 1000–5000); black lines additionally indicate genes referred to in the text. Green color marks those genes, whose transcripts are most abundant (NPKM values >5000) throughout all sampling points, whereas genes with highly abundant transcripts (NPKM values >5000) only at specific sampling points are shown in orange, red and blue. The most abundant transcript was assigned to *lanA2* in sample R-III. This gene encodes a component of the two-peptide lantibiotic lichenicidin [33] and is transcribed with NPKM values >5000 at all sampling points. Accordingly high transcript abundance can also be observed for *lanA1*, which codes for the second prepeptide of this lantibiotic. Further genes with similar abundances are coding for the BsrG-like peptide (BLi05015) [25,34], the sporulation protein SpoVG, the DNA-binding protein Hbsu, BLi01059 and Veg [35]. The gene encoding the oxygen detoxification protein SodA, the cold shock responsive genes *cspB* and *cspD*, the gene for the elongation factor Tu, and genes coding for components of the translation machinery are transcribed with NPKM values >1000 at all sampling points. Transcripts which are highly abundant exclusively in the later, productive stages of the process are associated to spore formation, whereas transcripts which are highly abundant during the early stages of the process encode ribosomal proteins, proteins of the TCA cycle and ATP synthase subunits. For an illustration of highly abundant proteins please refer to Additional file 1: Figure S8.

Previously, we curated the annotation of 4172 protein-coding genes and determined the respective transcript abundances in all samples [25,29], resulting in NPKM values from 0 for lacking transcripts to 85.267 for the most abundant transcripts (Figure 2). Analysis of the obtained data with baySeq [30] and ANOVA revealed that 980 and 1016 genes, respectively, are differentially expressed at the different sampling points. In total, 1395 genes were determined as differentially expressed by at least one method and utilized for further analysis. Generic GO slim enrichment analysis [31,32] revealed that genes assigned to regulation, protein modification and metabolism, DNA metabolism, cell cycle and translation are underrepresented within this dataset of differentially expressed genes. Overrepresented genes were assigned to protein transport, response to external stimuli, carbohydrate metabolic processes and cell differentiation.

**Figure 3:**
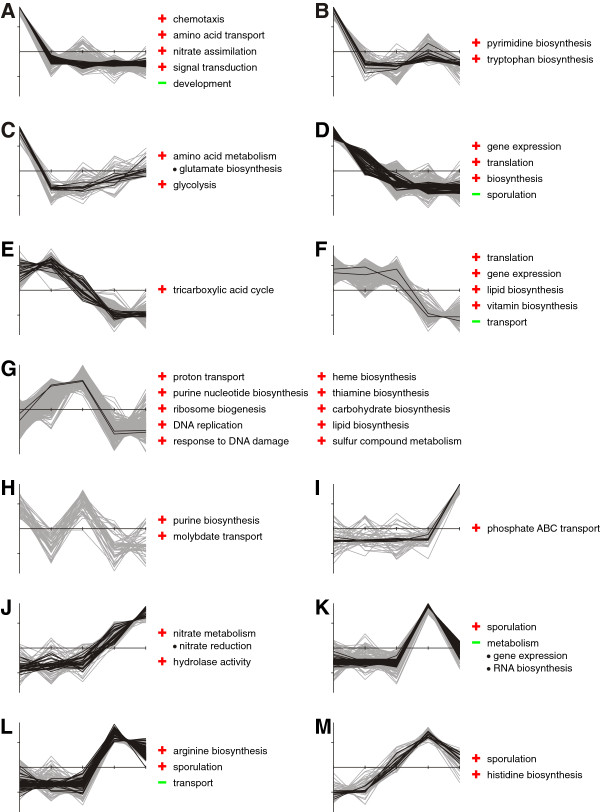
***k*-means clustering of expression profiles with assigned GO terms.** Thirteen gene clusters determined by *k*-means clustering are depicted with corresponding, significantly enriched GO terms [32] (see also Figure 4). Over- and underrepresented GO terms are indicated by red and green symbols, respectively. Black lines mark genes with a baySeq [30] likelihood value >0.99, which indicates differential expression; all other genes are colored in grey. All values are depicted by Z-score transformed NPKM values (y-axis) versus sampling points I to V (x-axis).

**Figure 4:**
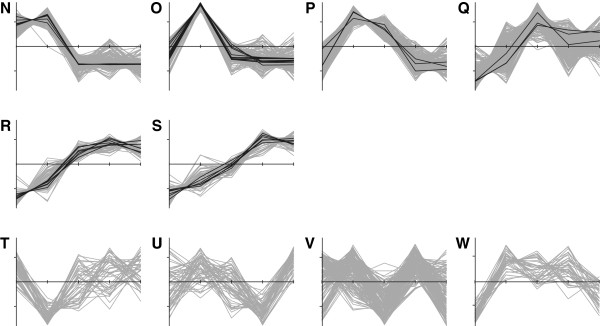
***k*-means clustering of expression profiles.** Ten gene clusters determined by *k*-means clustering with no significantly enriched GO terms (see also Figure 3). Black lines mark genes with a baySeq [30] likelihood value >0.99, which indicates differential expression; all other genes are colored in grey. All values are depicted by Z-score transformed NPKM values (y-axis) versus sampling points I to V (x-axis).

The transcriptome data allowed the assignment of 3567 genes to 23 clusters by *k*-means cluster analysis based on the determined differentially expressed genes (Figure 3 and Figure 4; Additional file 2: Table S1) [36]. Each cluster was also examined for over- and underrepresented groups of genes by GO term-based enrichment analysis (Additional file 2: Table S2) [32,37]. Clusters A-H and N-Q comprise genes which are more abundant at the early stages of the process than at the later sampling points. In this group, overrepresented genes are mainly involved in gene expression and translation, biosynthetic processes, transport and metabolism of amino acids, or central carbon metabolism including glycolysis and TCA cycle. Another pattern can be found for clusters I-M and R + S which contain genes displaying higher transcript abundances in the productive stage of the fermentation (sampling points IV and V). In clusters with the highest measured transcript abundances at stage IV (K-M) genes were predominantly involved in sporulation processes. Transcripts more abundant in stage V than in stage IV are depicted in clusters I and J and, among others*,* encompass genes for phosphate ABC transporter PstABC and nitrate reductase NarGHIJ.

Detailed analyses of transcript and protein abundances (Additional file 2: Table S3) concerning important factors of bacterial growth and productivity (amino acid transport and metabolism, central carbon metabolism, starvation and stress responses, and protein secretion) will be presented in the following passages.

### Amino acid transport and metabolism

**Figure 5:**
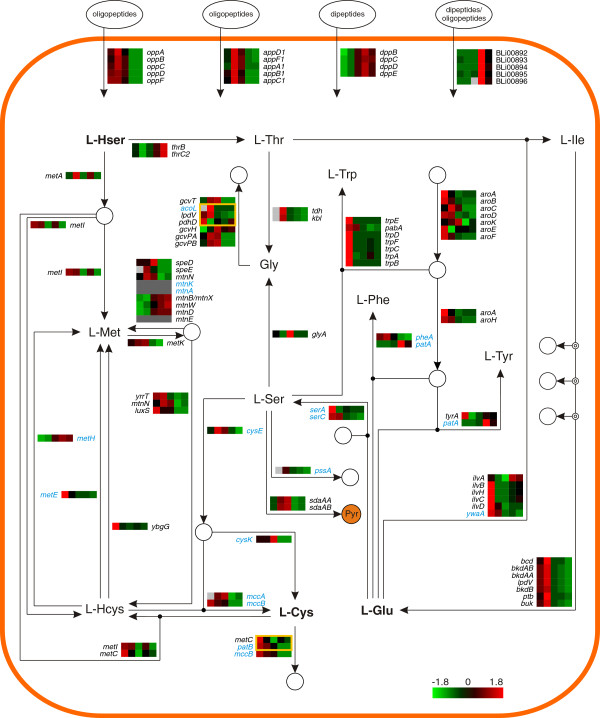
**Transcriptome of the amino acid metabolism - Part I.** Heat map representation of Z-score transformed NPKM values of genes involved in amino acid transport and metabolism (see also Figure 6). Genes with an assigned antisense RNA [25] are marked in blue, genes with NPKM values <10 at all sampling points are indicated by dark grey boxes and statistically not significant values are indicated by light grey boxes. Amino acids written in bold can also be found in Figure 6. Yellow frames indicate reactions with multiple assigned enzymes of which only one is strictly necessary. Pyr: Pyruvate. For the corresponding proteome data please refer to Additional file 1: Figure S2.

**Figure 6:**
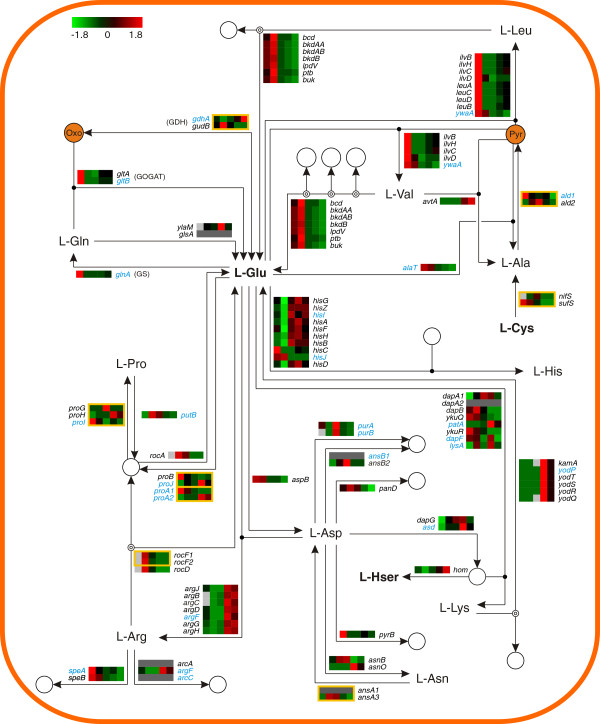
**Transcriptome of the amino acid metabolism - Part II.** Heat map representation of Z-score transformed NPKM values of genes involved in amino acid transport and metabolism (see also Figure 5). Genes with an assigned antisense RNA [25] are marked in blue, genes with NPKM values <10 at all sampling points are indicated by dark grey boxes and statistically not significant values are indicated by light grey boxes. Amino acids written in bold can also be found in Figure 5. Yellow frames indicate reactions with multiple assigned enzymes of which only one is strictly necessary. Pyr: Pyruvate, Oxo: 2-Oxoglutarate. For the corresponding proteome data please refer to Additional file 1: Figure S3.

The examined fermentation process was performed in the presence of a complex nitrogen source initially supplemented with glucose. To elucidate how *B. licheniformis* utilizes the supplied peptide substrate, the transcript (Figure 5 and Figure 6) and protein (Additional file 1: Figure S2 and Figure S3) abundances of the major amino acid metabolism-related genes were examined in the context of their metabolic network.

The genome of *B. licheniformis* encodes six unambiguous operons encoding peptide ABC transporters (*app1*, *app2*, *dpp*, *opp*, BLi00892-96, BLi02527-31) [25,29], four of them showing transcript abundance under the examined conditions (Figure 5). The *app1* and the *opp* operon each encode oligopeptide ABC transporter systems. They are transcribed during all stages of the fermentation process, but show top transcript abundances at the earlier sampling points, particularly at sampling point II (NPKM values >500). In contrast, the *dpp* operon encoding a dipeptide ABC transporter displays increased RNA abundance over time with maximal levels at sampling point IV. Furthermore, transcripts of the dipeptide/oligopeptide ABC transporter operon BLi00892-96 are only abundant at the later fermentation stages. Regarding the RNA abundances of the *opp* and *dpp* operons, similar patterns of activation and repression during cell growth and sporulation have been observed in *B. subtilis*[38-40]. In contrast, the *app1* operon, which is orthologous to the *app* operon of *B. subtilis*, does not resemble the sporulation-dependent regulation in the model organism [39,40], indicating a different regulation of this operon in *B. licheniformis.*

The RNA abundances of the ABC transporter operons seem to reveal a fermentation stage-dependent pattern, promoting the idea that oligopeptides are imported primarily, whereas dipeptides are presumably consumed after oligopeptides are exhausted. This transcriptional pattern may be influenced by the fact that dipeptides should become more available over time due to the activity of extracellular protease secreted within the fermentation process. The RNA abundances of further amino acid transporters are shown in Additional file 1: Figure S4.

#### Sampling point I

At this fermentation stage of carbon excess (Figure 1), transcripts of the operon of the glutamate synthase (GOGAT) *gltAB* and the gene of the glutamine synthetase (GS) *glnA* are highly abundant (Figure 6). This prompts the conclusion of a strong glutamate production, which is fed by 2-oxoglutarate provided by the catabolism of glucose (see *Central carbon metabolism*). The high transcript abundance of genes involved in the glutamate-dependent anabolism of proline, aspartate, alanine and aromatic and branched-chain amino acids (Figure 5 and Figure 6) indicates that the produced glutamate is utilized for the synthesis of other amino acids [41,42], despite the given complex amino acid broth.

Further active genes have been assigned to aspartate degradation for pyrimidine biosynthesis (Figure 3), arginine and S-adenosyl methionine (SAM) metabolism for putrescine synthesis, and cysteine degradation releasing sulfur-containing compounds (Figure 5 and Figure 6).

#### Sampling point II

Upon glucose exhaustion (Figure 1), the transcriptome indicates drastic changes in the fluxes of the amino acid metabolism. Transcripts of genes for glutamate-releasing catabolic processes are highly abundant, as it can also be observed for transcripts of genes promoting the degradation of proline, arginine and branched-chain amino acids (Figure 5 and Figure 6). In reverse, the transcripts of the glutamate-consuming pathways abundant at sampling point I have declined. Glutamate now seems to be metabolized to 2-oxoglutarate by the glutamate dehydrogenase (GDH) GudB and channeled into the TCA cycle. Complementarily, the transcripts of GS and GOGAT are also less abundant [41]. Furthermore, the observed transcript abundances indicate that threonine is metabolized to glycine which is then degraded by the glycine cleavage complex, in order to gain reducing equivalents and C1 compounds while serine is degraded to pyruvate by L-serine dehydratase SdaAAAB to provide further energy sources.

#### Sampling point III

During the later glucose exhaustion stage (Figure 1) most genes involved in amino acid metabolism show reduced transcript abundances. Elevated abundances are nearly exclusively found in pathways involved in serine degradation, such as the above mentioned conversion of serine to pyruvate, the metabolization to glycine for subsequent degradation by the glycine cleavage complex, and the conversion to cysteine which is then further metabolized to pyruvate via the intermediate alanine (Figure 5 and Figure 6).

The transcript abundances of *purA* and *purB* – and other genes associated with purine biosynthesis (Figure 3) - indicate the degradation of aspartate in order to provide building blocks for this pathway.

#### Sampling points IV & V

In the productive stages of the fermentation process (Figure 1), the determined RNA abundances show that the amino acid metabolism has progressed to the glutamate-consuming synthesis of proline and the nitrogen-rich amino acids arginine and histidine (Figure 6). The reason for this reaction may lie in the previous high induction of genes mediating the degradation of proline and arginine during the earlier stage of glucose exhaustion. The glutamate required for these anabolic reactions is delivered by the glutamate dehydrogenase GdhA [43], and the GOGAT/GS system which becomes slightly re-induced upon amino acid consumption and the applied glucose feed [41].

Further pathways whose transcripts are abundant at these fermentation stages include the synthesis of threonine via the anabolism of homoserine, and the conversion of valine to alanine (Figure 5 and Figure 6). Also, the transcripts of genes for the degradation of lysine are highly abundant; as members of the σ^E^ regulon, they are activated by the initiation of sporulation [44]. In addition to the high transcript abundance of sporulation-related genes shown in Figure 3, this is evidence for active sporulation within the fermenter population.

Of course, the conditions in the fermenter do not cause a response to nitrogen limitation as described by Voigt et al. [21]. However, the shut-down of branched-chain amino acid degradation during the phase of glucose exhaustion at sampling point III might be accounted to a limitation effect, as in *B. subtilis* the orthologous transcriptional regulator for activation of this pathway is induced by the presence of such amino acids [45]. Additionally, as transcripts of several amino acid synthesis pathways of are abundant during the later fermentation stages, these amino acids are seemingly not available in excess.

### Central carbon metabolism

**Figure 7:**
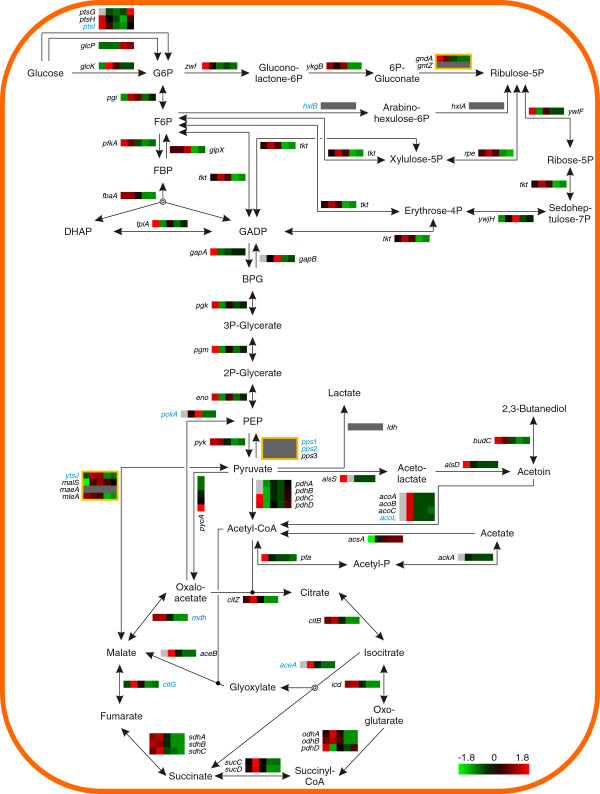
**Transcriptome of the central carbon metabolism.** Heat map representation of Z-score transformed NPKM values of genes involved in central carbon metabolism. Genes with an assigned antisense RNA [25] are marked in blue, genes with NPKM values <25 at all sampling points are indicated by dark grey boxes and statistically not significant values are indicated by light grey boxes. Yellow frames indicate reactions with multiple assigned enzymes of which only one is strictly necessary. For the corresponding proteome data please refer to Additional file 1: Figure S5.

The production process was initially supplemented with glucose. Upon depletion of this sugar and its derivates, a pulsed glucose feed was established in order to enhance the available energy. Thus, enzymes relevant for sugar catabolism (Figure 7; Additional file 1: Figure S5) and sugar transport (Additional file 1: Figure S6) are required to maintain an optimal energy supply throughout the fermentation. The transcriptional changes of those enzymes were analyzed at the different sampling points and will be described in the following passages.

#### Sampling point I

Shortly before the total depletion of the initially supplied glucose (Figure 1), the genes for glycolytic enzymes are highly transcribed (NPKM values from 359 to 2473). High transcript abundance has also been recorded for the genes of the oxidative pentose phosphate pathway and the TCA, but not for the embedded glyoxylate bypass [29] (Figure 7). Furthermore, the *alsSD* operon for acetoin synthesis and the phosphate acetyltransferase gene *pta* for acetate production were maximally transcribed, indicating the channeling of carbon to the production of overflow metabolites (Figure 1) [46]. However, varying transcript abundances of the acetate kinase gene (*ackA*), also involved in acetate synthesis, were observed. This is due to slightly asynchronous samples for this fermentation stage (Figure 1; Additional file 1: Figure S1) and restricts the determination of reproducible NPKM values for this gene and sampling point (Figure 7).

#### Sampling point II

After exhaustion of the carbohydrate source (Figure 1), the transcript abundances of the genes of acetate and acetoin synthesis decline (NPKM values <72) (Figure 7). This regulatory effect can be explained by the fact that the expression of the acetate synthesis genes *pta* and *ackA* is influenced by CcpA triggered carbon catabolite activation, as shown for *B. subtilis*[47-49], which ceases with glucose depletion. Furthermore, the acetoin synthesis operon *alsSD* is activated by the transcriptional regulator AlsR in the presence of acetate [50,51]. Therefore, it exhibits reduced transcription when acetate concentration decreases due to the dissimilation of the products formed during overflow metabolism (Figure 1). The dissimilatory reaction is caused by the termination of carbon catabolite repression, allowing an increasing transcript abundance of *acsA* (Figure 7), which encodes an acetyl-CoA synthetase for the conversion of acetate to acetyl-CoA [52]. Transcript abundance of the *acuABC* operon, which has been shown to lead to in- and reactivation of AcsA in *B. subtilis*[53,54], is also increased upon cessation of carbon catabolite repression (Additional file 1: Figure S7). However, an influence of this operon on the acetate or acetoin metabolism of *B. licheniformis* has not been revealed [55]. Furthermore, the transcript abundance of the *acoABCL* operon has strongly increased (NPKM values >2500) (Figure 7). The expression of the corresponding transcriptional activator gene, *acoR*, depends on induction by acetoin [55,56]. Therefore, a high concentration of acetoin is indicated by the high transcript abundance of this operon. In contrast, the gene of the acetoin reductase/2,3-butanediol dehydrogenase *budC* is only weakly transcribed (NPKM value <60) throughout the production process. Thus, it appears that no significant 2,3-butanediol production occurred under the given conditions.

Negative regulatory effects could be observed for the genes of the *gapA* and the *pdh* operon (Figure 7), which are repressed as reaction to glucose starvation [21]. In contrast, the genes coding for the isocitrate lyase AceA and the malate synthase AceB, reach their top level of transcript abundances at this sampling point. Both genes belong to the glyoxylate bypass, allowing *B. licheniformis* not only to gain energy by C2 compound oxidation, but also to grow on acetate and acetoin as sole carbon sources by bypassing the oxidative, CO_2_ evolving steps of the TCA cycle [55,57]. Additionally, the high transcript abundance of the other genes of the TCA cycle enables the utilization of 2-oxoglutarate provided from amino acid catabolism (see *Amino acid transport and metabolism*).

In general, the registered changes in metabolism during this process stage are in good accordance with results presented by a previous study on glucose starvation in *B. licheniformis*[21]. However, this is the first time that expression of these production-relevant genes [3] is shown during growth of *B. licheniformis* in rich medium.

#### Sampling point III

At this stage of the fermentation process, the C2 compounds were completely depleted and the cells entered a short phase of reduced metabolic activity (Figure 1). The genes coding for glycolytic enzymes also involved in gluconeogenesis, which have shown decreased transcript abundances at sampling point II, are slightly increased (Figure 7). This is confirmed by the amount of the corresponding proteins (Additional file 1: Figure S5). Furthermore, transcripts of exclusively gluconeogenic genes *gapB* and *glpX* show their maximal abundances at this sampling point (Figure 7). Phosphoenolpyruvate (PEP), the building block for gluconeogenesis, seems to be converted from oxaloacetate, as the gene for the phosphoenolpyruvate carboxykinase PckA is maximally transcribed. Contrarily, the genes for the phosphoenolpyruvate synthases Pps1, Pps2 and Pps3 [25], promoting PEP synthesis from pyruvate, show only low levels of transcript abundance (NPKM values <25). Additionally, the genes of the non-oxidative pentose phosphate pathway show their highest transcript abundances during the phase of glucose starvation (sampling point II and III). This regulatory effect is in accordance with previous observations in *B. licheniformis*[21], and is remarkable as no glucose-dependent regulation of this pathway has been found in *B. subtilis*[58].

Taken together, the observations indicate that the C2 compounds catabolized via the glyoxylate bypass are utilized for the generation of glucose and other sugars.

#### Sampling points IV & V

The last two samples were taken during the subtilisin production stage of the fermentation process. At these sampling points, glucose was added to the fermenter in pulsed feeding steps and channeled to energy metabolism via glycolysis and TCA cycle (Figure 7). Although the RNA abundances of both pathways are reduced compared to the previous sampling points, they are still abundant (NPKM values >100). Similar results were also obtained for the transcript abundances of genes involved in gluconeogenesis and the non-oxidative pentose phosphate pathway. These findings, together with the above described glutamate-consumption by anabolic amino acid pathways during the late stages of the fermentation process, indicate that the pulsed supply with glucose during this fermentation stage is not only sufficient for provision of reducing equivalents, but also for facilitation of 2-oxoglutarate formation needed for glutamate synthesis.

### Starvation and stress responses

Bacterial cells react to declining nutrient concentrations or changing environmental conditions by exhibiting well orchestrated starvation or stress responses. To elucidate whether *B. licheniformis* suffers of any of these situations during the fermentation process, we compared the obtained transcriptomic and proteomic data to described starvation and stress responses of *B. licheniformis* and *B. subtilis*[23,24,59-62].

**Figure 8:**
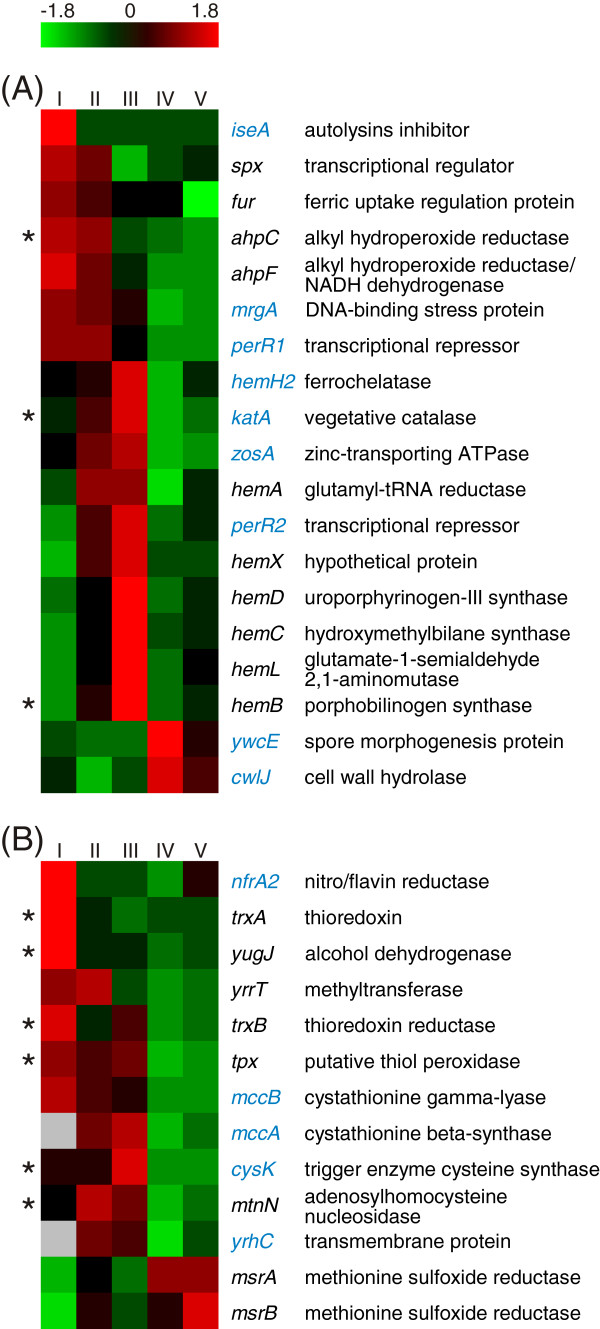
**Peroxide stress response.** Heat map representation of Z-score transformed NPKM values. The depicted **(A)** PerR and **(B)** Spx regulons have been identified as *B. licheniformis* peroxide stress markers by Schroeter et al. [23]. Genes with an assigned antisense RNA [25] are marked in blue, asterisks indicate a detected protein spot for the respective gene (Additional file 2: Table S3) and statistically not significant values are marked by grey boxes.

In *B. licheniformis*, oxidative stress induced by hydrogen peroxide results in increased RNA abundances of the PerR, Spx, Fur, and SOS regulon [23]. In our study, we found the PerR as well as the Spx regulon (Figure 8) temporarily induced during the early stages of the fermentation process (sampling points I to III). The transcript as well as the gene product of the superoxide dismutase-encoding *sodA* were highly abundant at all sampling points (see also Figure 2 and Additional file 1: Figure S8), leading to an accumulation of the SodA protein over time. A similar pattern of transcript abundance and protein accumulation could also be observed for the putative thiol peroxidase Tpx, but not for the vegetative catalase KatA. In contrast to these results, the Fur regulon and the SOS regulon did not show distinct RNA abundances. In *B. subtilis*, the SOS regulon has been described to be more responsive to hydrogen peroxide than to superoxide exposure [59]. Therefore, and also considering the high transcript abundance of *sodA*, we infer a cellular response to a potentially toxic superoxide load at the early stages of the fermentation process.

Strikingly, we found the *lan* gene cluster [33,63], which encodes the genes for lantibiotic production and immunity, to show a high transcript abundance during the early stages of the fermentation process (Additional file 1: Figure S9). The highly abundant transcripts of the lichenicidin prepeptide genes *lanA2* and *lanA1* depicted in Figure 2 are members of this cluster. Although the transcript abundances decline at the later fermentation stages, they remain on a high level, indicating a substantial lichenicidin production during the fermentation process. In consistence with the elevated lichenicidin challenge, cell envelope stress responsive operons like *liaRSFGHI*[24] display transcript abundances during the early stages of the process (Additional file 1: Figure S10). In the following stages, the level of transcripts declines to NPKM values still >100; corresponding to the high levels of lantibiotic-coding mRNA over the complete fermentation process. It remains elusive why the cells channel energy to the production of antimicrobial compounds and the corresponding immunity response while grown in pure culture.

The performed cluster analysis indicated emerging transcript abundances for sporulation-dependent genes at sampling point IV (Figure 3; Additional file 1: Figure S11). In general, sporulation is observed as a complex, energy-consuming response to nutrient limitation, which is activated by the master regulator of sporulation Spo0A [64]. Obviously, sporulation is rather unproductive in terms of industrial fermentation and thus undesired during such processes. Unfortunately, it has been shown that deletion of Spo0A does not only result in a sporulation-deficient phenotype, but also in increased cell lysis [65]. In *B. subtilis*, it has been shown that activation of Spo0A is influenced by a cascade of different regulatory systems, including the potassium leakage-sensing kinase KinC [66,67]. Strikingly, one known effect of two-peptide lantibiotics like the aforementioned *lan* cluster is indeed the induction of potassium leakage [68,69]. Thus, our data indicate a lichenicidin-mediated sporulation induction within the fermentation. The deletion of the *lan* system may present a promising approach to strain optimization, as this should lead to reduced levels of phosphorylated Spo0A with less probability of exceeding the sporulation-inducing threshold.

A detailed inspection of iron starvation and heat shock response did not reveal any distinct activation patterns (Additional file 1: Figure S12 and Figure S13), whereas a notable phosphate starvation response could be identified at the latest stage of one fermentation (Additional file 1: Figure S14).

### Protein secretion

**Figure 9:**
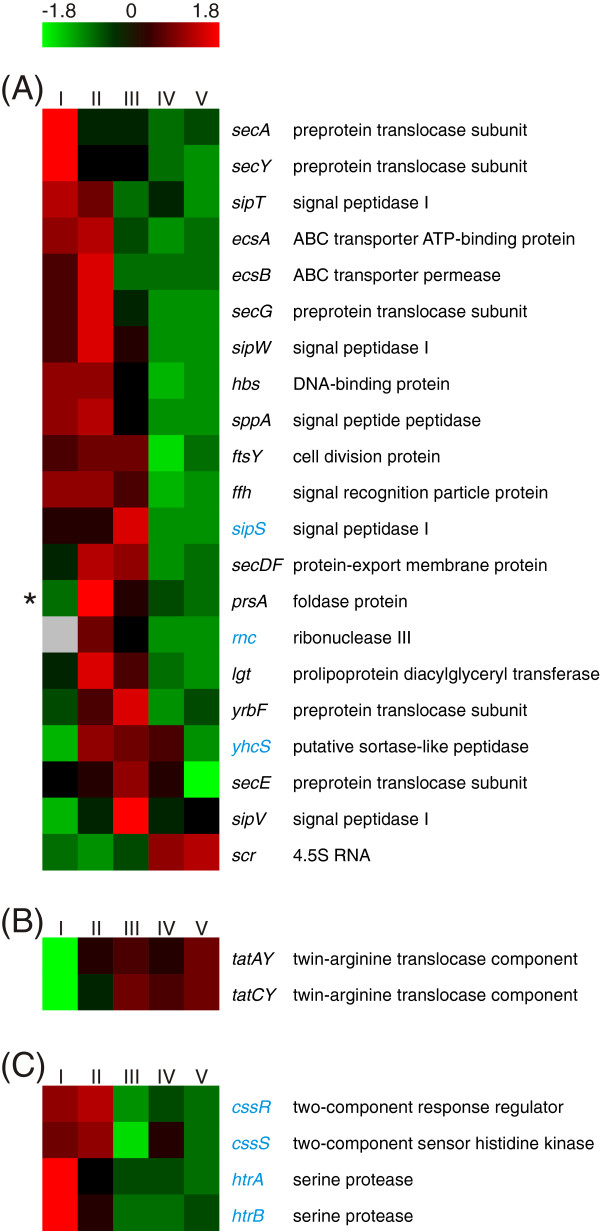
**Secretion.** Heat map representation of Z-score transformed NPKM values. The depicted genes belong to **(A)** the Sec pathway, **(B)** the Tat pathway and **(C)** the secretion stress CssR regulon [73,76-79]. Genes with an assigned antisense RNA [25] are marked in blue, asterisks indicate a detected protein spot for the respective gene (Additional file 2: Table S3) and statistically not significant values are marked by grey boxes. The genes *tatAD* and *tatCD* are not shown due to lacking transcript abundances (NPKM values <10).

In the Gram-positive model organism *B. subtilis,* secretion of subtilisin is directed via the secretory (Sec) pathway [14]. Subtilisin is synthesized as preproenzyme [70] containing a Sec-dependent signal peptide [14] and a propeptide, which serves as intramolecular chaperone [71]. The nascent protein chain is recognized by the signal recognition particle (SRP) and transferred to the membrane [72] where it is forwarded to the Sec translocase and transported across the membrane [73]. After cleavage of the signal peptide, the subsequent protein folding into pro-subtilisin is aided by the propeptide [74] and the extracytoplasmic chaperone PrsA [75]. Following the autoprocessed cleavage of the propeptide, it is degraded *in trans* and the active enzyme is released into the extracellular space [71]. The heat map depicted in Figure 9A shows the RNA abundances of the required orthologous genes in *B. licheniformis*. Interestingly, the transcript abundances of these genes decline in the late stages of the process in which the main amount of subtilisin is secreted. This observation is also supported on protein level by the abundance of PrsA, which declines at the later sampling points (Additional file 2: Table S3). The only exception to this pattern is the highly abundant SRP component *scr* (4.5S RNA) which shows increased RNA abundance at the later sampling points.

In contrast to genes of the Sec pathway, the transcript abundances of the genes of the twin-arginine translocation (Tat) system TatAyCy double from sampling point I to II (Figure 9B) and increase further to maximal abundance (NPKM values 1221 and 1155) at the latest sampling point. In contrast to TatAyCy, transcripts of the second Tat system TatAdCd do not show any abundance during the fermentation process. Five proteins were predicted to contain a corresponding Tat signal peptide. However, the pattern of transcript abundances of these proteins does not indicate that they are the main secretion targets for the strongly transcribed TatAyCy system. It has recently been shown that the extracellular *B. subtilis* lipase BSU02700, which is Sec-dependently secreted under standard conditions, is translocated by the *B. subtilis* Tat pathway in a hyper-secreting strain [80]. Hence, this phenomenon has been assumed to be an overflow mechanism [80]. Considering the RNA abundances shown for the secretion machinery in this study (Figure 9), it is tempting to speculate that this overflow mechanism may also play a role in the secretion of subtilisin in *B. licheniformis*. The fact that no typical Tat signal peptide is attached to the subtilisin proenzyme seems to argue against this hypothesis. However, it was shown that the conservation of the RR motif of the signal peptide [81] is not essential for Tat-dependent secretion [82,83]; an RK motif, as present in the Subtilisin Carlsberg prepeptide, can likewise facilitate Tat-dependent secretion (Additional file 1: Figure S15) [82,83]. The blurred boundaries of Tat- and Sec-dependent secretion are additionally pointed out by the facts that proteins with a Tat signal peptide can, *vice versa*, be secreted by the Sec pathway and that the Tat system is accessible for originally Sec-dependent proteins fused to Tat signal peptides [84,85]. This was demonstrated by the detection of TatAyCy-dependent export of active subtilisin [85] and gives reason to assume that Tat-dependent subtilisin secretion is not an obstacle for proper folding of the enzyme.

In *B. subtilis*, a CssRS-dependent response to protein secretion stress triggered by both, homologous and heterologous proteins, has been described [86-88]. The two-component regulatory system CssRS reacts to secretion stress by activating the transcription of *htrA* and *htrB*[87], which encode membrane-anchored serine proteases that trigger refolding or degradation of misfolded or aggregated proteins [89]. The here determined RNA abundances of the genes of the CssR regulon are given in Figure 9C. High transcription rates can be observed for the genes of the serine proteases HtrA and HtrB at the first sampling point, but these rates decline at later stages of the process. This reaction could be due to various reasons: (i) even highly synthesized pre-subtilisin does not aggregate in the cells, (ii) the cells are highly tolerant to large amounts of (aggregated) pre-subtilisin or (iii) the preprotein is efficiently exported in the late production stages, maybe even supported by the Tat pathway.

## Conclusion & outlook

The presented data give unprecedented insights into the complex adaptations of the bacterial production strain *B. licheniformis* DSM13 to the changing physiological demands during an industrial-oriented fermentation using the example of a detergent protease production process. We thereby provide reference data for a better understanding and possible optimization of industrial fermentation processes.

These insights enabled us to pinpoint physiological adaptations within the bioprocess, many of which could be confirmed by proteome analysis. Cluster analysis clearly revealed strong growth phase dependencies of many genes as well as some phase-independent genes. RNA as well as protein abundances of the central carbon metabolism and the amino acid metabolism are in accordance with the initial glucose-driven metabolism. Main changes in the corresponding pathways regard the overflow metabolism and the subsequent catabolism of the thereby produced C2 compounds as well as the alternating synthesis and degradation of glutamate and its derivates. Changes in RNA abundances reflect a transition of sustenance from more complex molecules like peptides and oligomers to amino acids. This emphasizes the importance of the secreted protease and its activity on the substrate as a functional component of the productive fermentation.

By comparing our data to previous transcriptome studies focusing on stress conditions, we were able to reliably identify potential stress factors within the process. A detailed inspection of the associated transcripts revealed oxidative stress and increasing phosphate limitation as important factors. Notably, the transcripts of the lichenicidin biosynthesis-related genes *lanA1* and *lanA2* are highly abundant throughout all sampling points. The high abundances of the complete *lan* gene cluster and the majority of sporulation-involved genes indicate a substantial production of antimicrobial compounds and a responsive, KinC-enhanced induction of sporulation. This energy-consuming behavior is clearly not supportive in terms of productivity.

An interesting finding concerning protein secretion pathways is the increase in abundance of the Tat pathway components *tatAY* and *tatCY* from sampling point I to V, which is in contrast to the abundance patterns of genes of the Sec secretory system. Thus, the cells seem to increase the overall secretion capacity during the production process by including non-typical secretory pathways.

The presented findings enabled the identification of important physiological and genetic switches of *B. licheniformis* which limit the overall productivity. The data indicate several opportunities to improve the strains performance in the production of subtilisin. The observed adaptions to the changing substrate supply during the successive metabolization of media components suggest that an optimization of the non-optimal amino acid composition or phosphate supply may lead to better reproducibility, increased efficiency, cycle time reduction, and finally a diminished employment of resources. Optimization of the deployed strain should also be achieved by the introduction of genetic modifications. For instance, the observed strong expression of the *lan* gene cluster which encodes an undesirable cell wall stress inducing byproduct marks it as promising target for a gene deletion. Another approach might be the modulation of the subtilisin signal peptide to channel subtilisin to the putative Tat-dependent secretion pathway.

## Methods

### Bacterial strains and fermentation

The samples for the proteome analysis were derived from fermentation experiments carried out for *Bacillus licheniformis* MW3Δspo described earlier. For detailed description of fermentation conditions, sampling points and sequencing of the transcriptome please refer to Wiegand et al. [25].

### Preparation of cytosolic protein extracts

50 mL of harvested cells were supplemented with 0.5 mL of protease inhibitor (3758.1, Carl Roth, Germany) directly upon sampling. Centrifugation was carried at 4500× *g* and 4°C for 10 min. The supernatant was removed and the cells were stored at -80°C.

For preparation of the cytosolic protein extracts the insoluble components of the fermentation medium were removed from the bacterial pellet by washing at least three times in ice cold 100 mM Tris/HCl, pH 7.5 buffer at 10000× *g* and 4°C for 10 min. After the last washing step the pellet was resuspended in 600 μL TE buffer (10 mM Tris/HCl, pH 7.5, 1 mM EDTA) containing 1.4 mM phenylmethylsulfonyl fluoride (PMSF). After addition of 250 μL glass beads (0.25-0.5 mm) the cells were disrupted by using RiboLyser cell mill (30 s at 6800 rpm, 5 min on ice, 30 s at 6800 rpm; Hybaid, UK). Glass beads and cell debris were removed by two centrifugation steps at 13000× *g* and 4°C for 30 min. To remove ions, which could disturb the isoelectric focusing, the protein extracts were purified with Amicon Ultra 3 K Centrifugal Filters (Millipore, Germany). The protein concentration of the protein extracts was determined using Roti®NanoQuant (Carl Roth, Germany).

### Two dimensional gel electrophoresis, imaging and quantification

Isoelectric focusing (IEF) of the cytosolic protein extracts was performed according to [90]. IPG *Blue*Strips 4–7 (SERVA, Germany) were loaded with 150 μg protein extract, which was adjusted to 340 μL with 2 M thiourea/8 M urea buffer and 34 μL CHAPS solution (20 mM DTT, 1% w/v CHAPS detergent) and rehydrated over night. IEF was carried out on a Multiphor II unit (Amersham Pharmacia Biotech) employing the following step gradient: 150 V for 150 Vh,300 V for 300 Vh, 600 V for 600 Vh, 1500 V for 1500 Vh, and a final phase of 3000 V for 57.5 kVh at 20°C. Before separation in the second dimension the IPG strips were incubated in 3.5 mL equilibration buffer A (2.4 M urea, 12% v/v glycerol, 4% v/v 0,5 M Tris pH 6.8, 55.5 mM SDS, 9 mM DTT) and equilibration buffer B (2.4 M urea, 12% v/v glycerol, 4% v/v 0,5 M Tris pH 6.8, 55 mM SDS, 9 mM DTT, 97 mM iodacetamide, 0.15 mM bromphenol blue), each for 15 min on an orbital shaker. Electrophoresis of the proteins was carried out on 12.16% acrylamide/0.34% bisacrylamide gels at 40 W for 1 h and 16 W for additional 16.5 h at 12°C. Gels were stained with Flamingo™ Fluorescent Gel Stain (Bio-Rad Laboratories, USA) following the manufacturer’s instructions. The gels were imaged with a Typhoon Imager 9400 (GE Health Care, UK). Spot detection was performed semi-manually with the Delta2D software version 4.1 (Decodon, Germany). Spot quantification was also done with the Delta2D software as described by Wolf et al. [91]. Quantities for proteins represented by more than one distinct spot are given for each spot separately.

### Identification of proteins from 2D gel spots

Selected protein spots were excised from 2-D gels using a spot cutter (Bio-Rad, USA). Digestion with trypsin and spotting on the MALDI-target was achieved using the Ettan Spot Handling Workstation (GE Health Care, UK) employing the manufacturers’ protocol. Mass spectrometry was carried out with a Proteome Analyzer 4800 (Applied Biosystems, USA) according to Wolf et al. [91]. The spectra were recorded in a mass range from 900 to 3700 Da with a focus mass of 1600 Da. An internal calibration was performed automatically when the autolytic fragments of trypsin with the mono-isotopic (M + H)^+^ m/z at 1045.556 or 2211.104 reached a signal to noise ratio (S/N) of at least 20.

Peak lists were created by using the script of the GPS Explorer TM Software Version 3.6 (Applied Biosystems, USA) with the following settings: mass range from 900 to 3700 Da, peak density of 15 peaks per 200 Da, minimal area of 100 and maximal 60 peaks per spot. The peak list was created for an S/N ratio of 15. MALDI-TOF-TOF measurements were carried out for the five strongest peaks of the TOF-spectrum. Using a random search pattern, 25 sub-spectra with 125 shots per sub-spectrum were accumulated for one main spectrum. The mono-isotopic arginine (M + H)^+^ m/z at 175.119 or lysine (M + H)^+^ m/z at 147.107 was used for internal calibration (one-point-calibration) when it reached a signal to noise ratio (S/N) of at least 5. Peak lists were created with the following settings: mass range from 60 to precursor – 20 Da, peak density of 15 peaks per 200 Da, minimal area of 100 and maximal 65 peaks per precursor. The peak list was created for an S/N ratio of 10. For data base search the Mascot search engine version 2.4.0 (Matrix Science Ltd, UK) with a specific *Bacillus licheniformis* (http://www.uniprot.org/uniprot/?query=Bacillus+licheniformis&sort=score) database was used.

### Statistical data analysis

NPKM (nucleotide activity per kilobase of exon model per million mapped reads) values [25] were computed for every protein-coding gene in all samples as a measure of RNA abundance. Based on the NPKM values analysis of differential expression was performed with baySeq [30] and one-way ANOVA [92]. Genes were assumed to be differentially expressed with a resulting baySeq likelihood value >0.9 or an ANOVA-based *p*-value <0.01 (False Discovery Rate (FDR) 2%).

### k-means clustering

*k*-means clusters were generated to identify fermentation stage-dependent trends in gene expression. (i) To ensure that the data of each replicate are sufficiently reliable, t-tests [93] were performed using TM4 MeV v4.8 software [36]. At least three out of the five samples had to have a *p*-value <0.15 to be taken into further analysis. (ii) For setting up the clusters only transcripts with baySeq likelihood values >0.99 were applied to the next step. (iii) Means of the replicates of each sampling point were calculated and z-score transformation was performed to gain a mean expression value of 0 and a standard deviation of 1 [94]. Clusters A to K and M to S were generated with TM4 MeV v4.8 [36] employing *k*-means clustering with Euclidian distances after estimating the cluster number by Figure of merit (FOM) analysis [94]. Clusters were cured manually. (iv) Finally, expression profiles of all other genes (*p*-value <0.15) were added to the determined clusters. Therefore Gene Distance Matrices were computed with TM4 MeV v4.8 [36] in Euclidian distance for all remaining transcripts and the respective cluster means as point of reference. Transcripts were assigned to the previously determined clusters dependent on their scaled distance value. In general the scaled distance value had to be <0.3, exceptions are clusters G and Q with a scaled distance value <0.6. 312 of the remaining 332 expression profiles could be assigned to the newly defined clusters L and T - W.

### GO

Gene Ontology terms [37] have been assigned to the genome of *B. licheniformis* using Blast2GO [32]. Enrichment analysis for every cluster was performed with the implemented Gossip [95] package running a two-tailed Fisher’s Exact Test (FDR 0.05). Go terms over- or underrepresented were sorted to their respective child or parent using OBO-Edit. To enable a broad overview of enriched groups Generic GO slims [31] also were assigned and analyzed with Blast2GO.

### Heat maps

Color codes presented in the heat maps are based on z-score transformed mean NPKM values for the transcriptome and mean spot quantities for the proteome. Figures 5, 6 and 7 were designed utilizing the CellDesigner™ v4.2 software [96] and employing the databases of SubtiWiki [76], BioCyc [97], KEGG [98] and IMG [99].

### Tat signal prediction

Proteins with RR and KR motifs of Tat signal peptides where predicted employing the TatP v1.0 software [100].

## Abbreviations

(M + H)+: Protonated molecular ions; °C: Degrees Celsius; μL: Microliter; 2D: Two dimensional; BPG: 1,3-Biphosphoglycerate; C2: Organic molecule harboring two carbon atoms; CHAPS: 3-[(3-Cholamidopropyl)dimethylammonio]-1-propanesulfonate; CoA: Coenzyme A; Da: Dalton; DHAP: Dihydroxyacetone phosphate; DTT: Dithiothreitol; F6P: Fructose 6-phosphate; FBP: Fructose 1,6-bisphosphate; FDR: False Discovery Rate; g: Gravitational constant; G6P: Glucose 6-phosphate; GADP: Glyceraldehyde 3-phosphate; GDH: Glutamate dehydrogenase; GO: Genome ontology; GOGAT: Glutamate synthase; GS: Glutamine synthetase; H: Hours; H2O2: Hydrogen peroxide; HCl: Hydrogen chloride; IEF: Isoelectric focusing; IPG: Immobilized pH gradient; kVh: Kilovolt hour; M: Molar; m/z: Mass-to-charge ratio; MALDI: Matrix-assisted laser desorption/ionization; min: Minute; mL: Milliliter; mM: Millimolar; mm: Millimeter; NPKM: Nucleotide activity per kilobase of exon model per million mapped reads; Oxo: Oxoglutarate; P: Phosphate; PEP: Phosphoenolpyruvate; Pyr: Pyruvate; RK motif: Twin-arginine motif; RNA-Seq: RNA sequencing; Rrpm: Revolutions per minute; RR motif: Arginine-lysine motif; s: Second; S/N: Signal-to-noise ratio; SDS: Sodium dodecyl sulfate; SRP: Signal recognition particle; Tat: twin-arginine translocation; TAXI: TRAP-associated extracytoplasmic immunity protein; TCA: Tricarboxylic acid; TOF: Time-of-flight mass spectrometer; TRAP: Tripartite ATP-independent periplasmic dicarboxylate transporter; V: Volt; v/v: Volume per Volume; Vh: Volt hour; W: Watt; w/v: Weight per volume.

## Competing interests

The authors declare that they have no competing interests.

## Authors’ contributions

SW performed the experiments, analyzed data and wrote paper, BV supervised proteomics experiments and analyzed data, DA performed mass spectrometry analyses, JB and SE provided industrial fermentation facilities and organized fermentation and sampling, MH granted access to facilities for proteome analysis, RD wrote paper and provided research facilities, HL wrote paper, designed research and analyzed data. All authors read and approved the final version of the manuscript.

## Supplementary Material

Additional file 1: Figures S1–S15 **Figure S1**: Protease production and process parameters. **Figure S2**: Proteome of the amino acid metabolism – Part I. **Figure S3**: Proteome of the amino acid metabolism – Part II. **Figure S4**: Amino acid transport. **Figure S5**: Proteome of the central carbon metabolism. **Figure S6**: Carbohydrate transport. **Figure S7**: Acetoin utilization operon *acuABC*. **Figure S8**: Most abundant proteins. **Figure S9**: Lichenicidin gene cluster. **Figure S10**: Cell envelope stress response. **Figure S11**: Sporulation. **Figure S12**: Iron starvation. **Figure S13**: Heat shock response. **Figure S14**: Phosphate stress response. **Figure S15**: Putative Tat signal peptide of Subtilisin Carlsberg. Click here for file

Additional file 2: Tables S1–S3 **Table S1**: *k*-means clustering of expression profiles. **Table S2**: GO term enrichment analysis of *k*-means clusters. **Table S3**: Proteome data. Click here for file
