# Supplementary material for: Fermentation stage-dependent adaptations of Bacillus licheniformis during enzyme production
Source: Microb Cell Fact. 2013 Dec 6;12:120. doi: 10.1186/1475-2859-12-120 (PMC3878961; doi:10.1186/1475-2859-12-120)
Supplement: Additional file 1: Figures S1–S15 — Figure S1: Protease production and process parameters. Figure S2: Proteome of the amino acid metabolism – Part I. Figure S3: Proteome of the amino acid metabolism – Part II. Figure S4: Amino acid transport. Figure S5: Proteome of the central carbon metabolism. Figure S6: Carbohydrate transport. Figure S7: Acetoin utilization operon acuABC. Figure S8: Most abundant proteins. Figure S9: Lichenicidin gene cluster. Figure S10: Cell envelope stress response. Figure S11: Sporulation. Figure S12: Iron starvation. Figure S13: Heat shock response. Figure S14: Phosphate stress response. Figure S15: Putative Tat signal peptide of Subtilisin Carlsberg. [file 1475-2859-12-120-S1.pdf]

Additional File 1

Fermentation stage-dependent adaptations of *Bacillus licheniformis* during enzyme production

Sandra Wiegand, Birgit Voigt, Dirk Albrecht, Johannes Bongaerts, Stefan Evers, Michael Hecker, Rolf Daniel and Heiko Liesegang

Content

Figure S1 Protease production and process parameters. 2
Figure S2 Proteome of the amino acid metabolism – Part I. 3
Figure S3 Proteome of the amino acid metabolism – Part II. 4
Figure S4 Amino acid transport. 5
Figure S5 Proteome of the central carbon metabolism. 7
Figure S6 Carbohydrate transport. 8
Figure S7 Acetoin utilization operon *acuABC*. 10
Figure S8 Most abundant proteins. 10
Figure S9 Lichenicidin gene cluster. 11
Figure S10 Cell envelope stress response. 12
Figure S11 Sporulation. 14
Figure S12 Iron starvation 22
Figure S13 Heat shock response. 23
Figure S14 Phosphate starvation response. 24
Figure S15 Putative Tat signal peptide of Subtilisin Carlsberg. 25
References 26

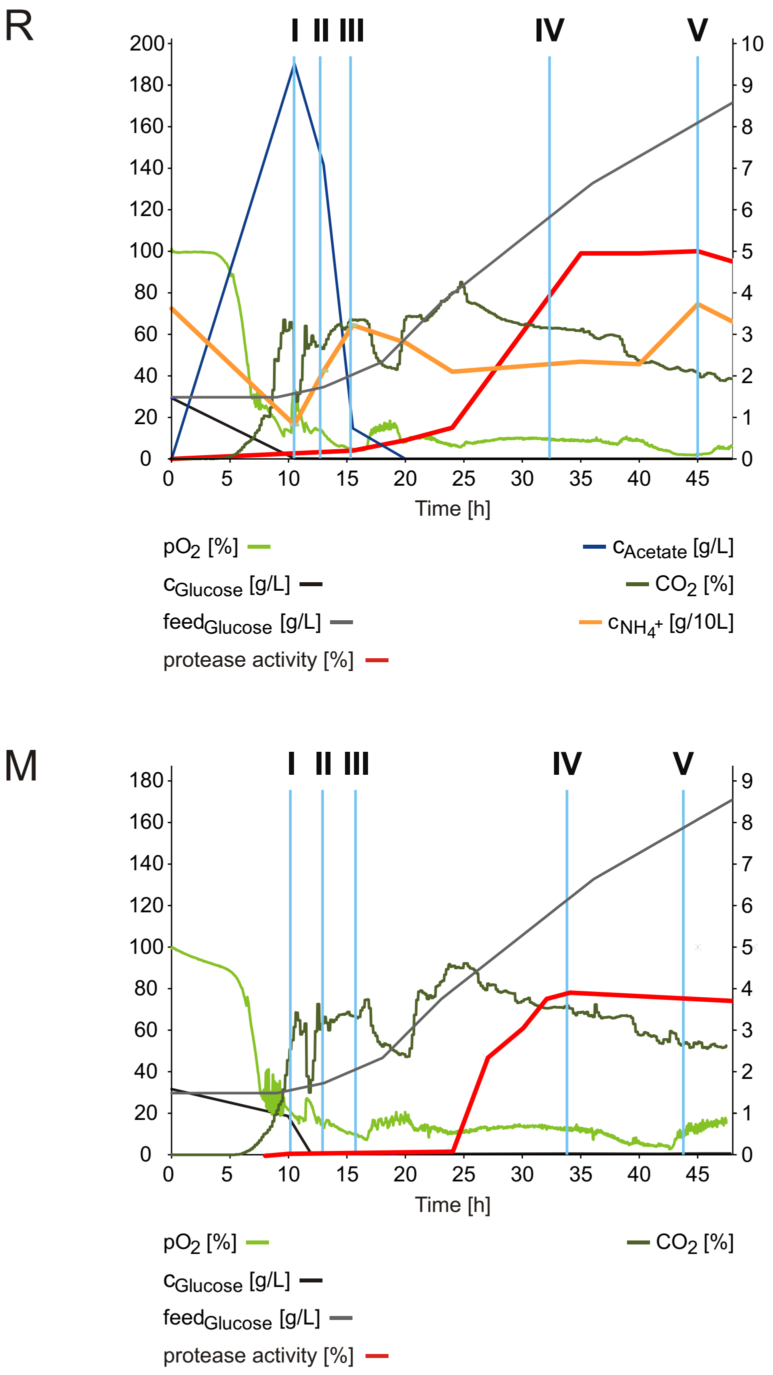

Figure S1 Protease production and process parameters. Process parameters are shown for fermentations R and M (please refer to Figure 1 for replicate L). Oxygen partial pressure pO_2 [%], glucose concentration $c_{Glucose}$ [g/L], supplied glucose $feed_{Glucose}$ [g/L] and normalized protease activity [%] are displayed on the left y-axis, whereas acetate concentration $c_{Acetate}$ [g/L] (only for fermentation R), carbon dioxide content CO_2 [%], and ammonium concentration c_{NH4+} [g/10L] (only for fermentation R) are scaled on the right y-axis. Process time t [h] is given on the x-axis. The sampling points I to V are indicated by light blue lines.

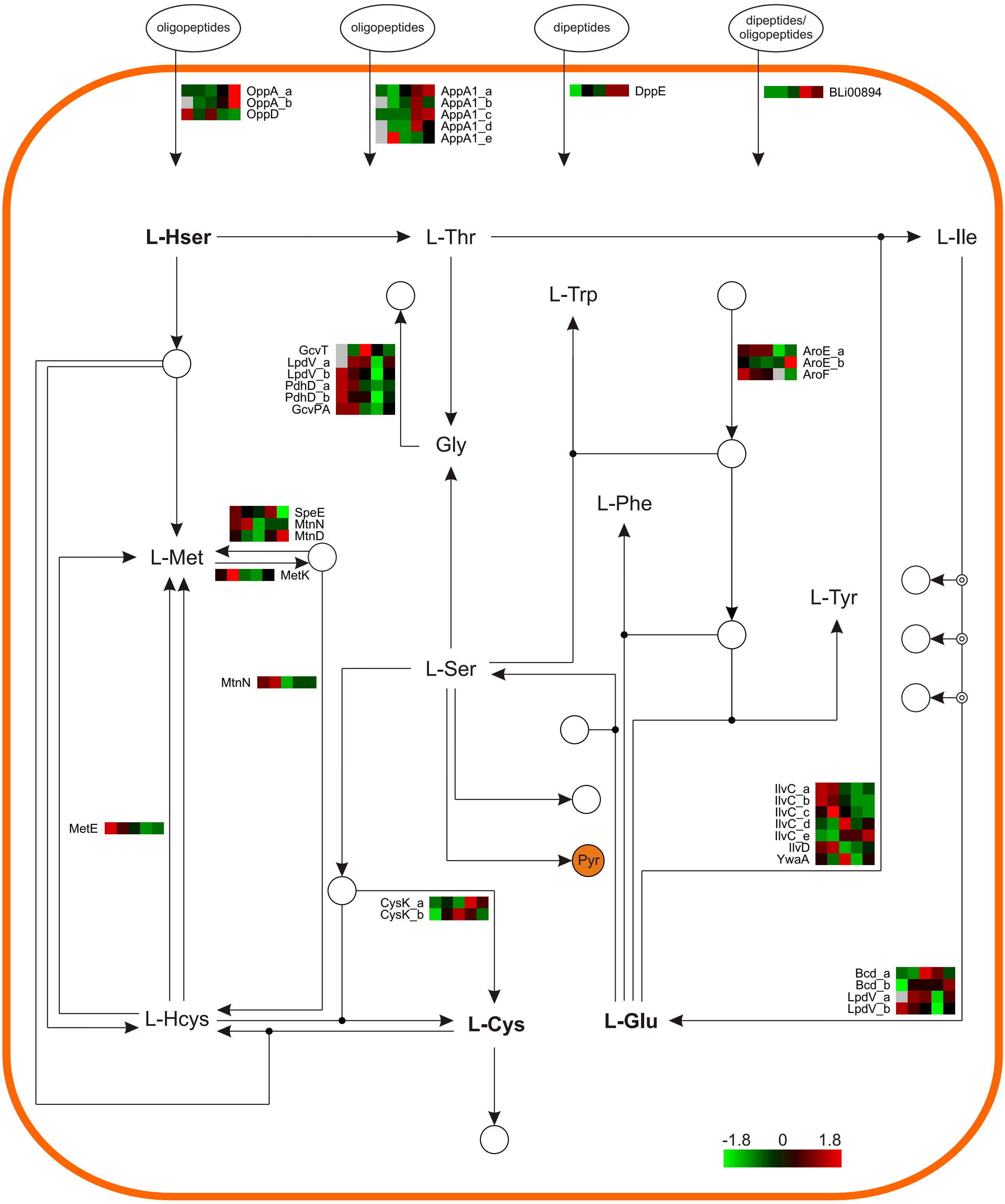

Figure S2 Proteome of the amino acid metabolism – Part I. Heat map representation of Z-score transformed protein spot volumes of proteins involved in amino acid transport and metabolism. In cases where a specific protein is assigned to more than one spot, the particular spots are indicated by an underscore, followed by an ordering letter. Statistically not significant values are indicated by light grey boxes. Pyr: Pyruvate. For the corresponding transcriptome data please refer to Figure 5.

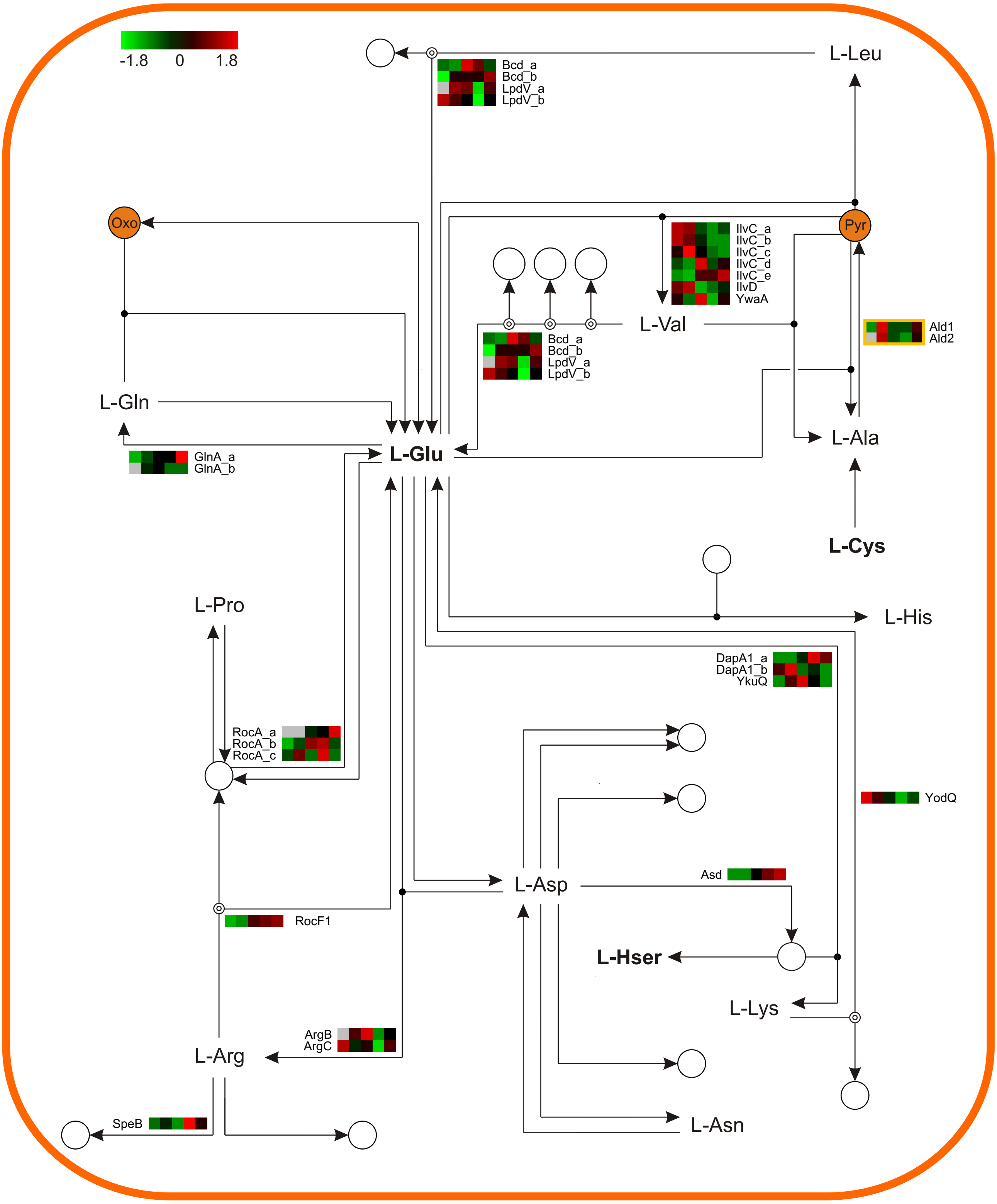

Figure S3 Proteome of the amino acid metabolism – Part II. Heat map representation of Z-score transformed protein spot volumes of proteins involved in amino acid metabolism. In cases where a specific protein is assigned to more than one spot, the particular spots are indicated by an underscore followed, by an ordering letter. Statistically not significant values are indicated by light grey boxes. Yellow frames indicate reactions with multiple assigned enzymes of which only one is strictly necessary. Pyr: Pyruvate, Oxo: 2-Oxoglutarate. For the corresponding transcriptome data please refer to Figure 6.

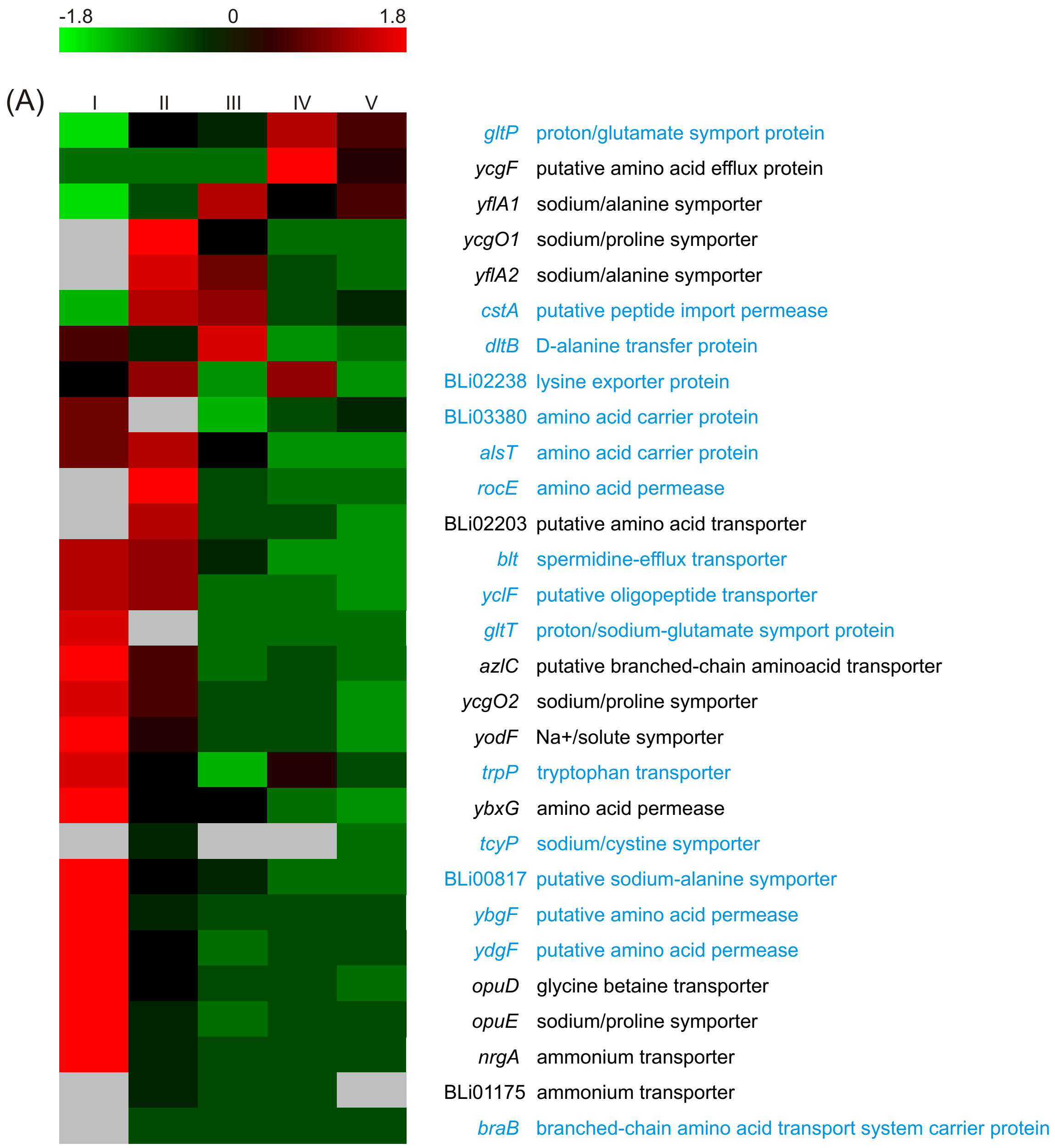
-1.8
0
1.8
(A)
I
II
III
IV
V
gltP proton/glutamate symport protein
ycgF putative amino acid efflux protein
yflA1 sodium/alanine symporter
ycgO1 sodium/proline symporter
yflA2 sodium/alanine symporter
cstA putative peptide import permease
dltB D-alanine transfer protein
BLi02238 lysine exporter protein
BLi03380 amino acid carrier protein
alsT amino acid carrier protein
rocE amino acid permease
BLi02203 putative amino acid transporter
blt spermidine-efflux transporter
yclF putative oligopeptide transporter
gltT proton/sodium-glutamate symport protein
azlC putative branched-chain aminoacid transporter
ycgO2 sodium/proline symporter
yodF Na+/solute symporter
trpP tryptophan transporter
ybxG amino acid permease
tcyP sodium/cystine symporter
BLi00817 putative sodium-alanine symporter
ybgF putative amino acid permease
ydgF putative amino acid permease
opuD glycine betaine transporter
opuE sodium/proline symporter
nrgA ammonium transporter
BLi01175 ammonium transporter
braB branched-chain amino acid transport system carrier protein

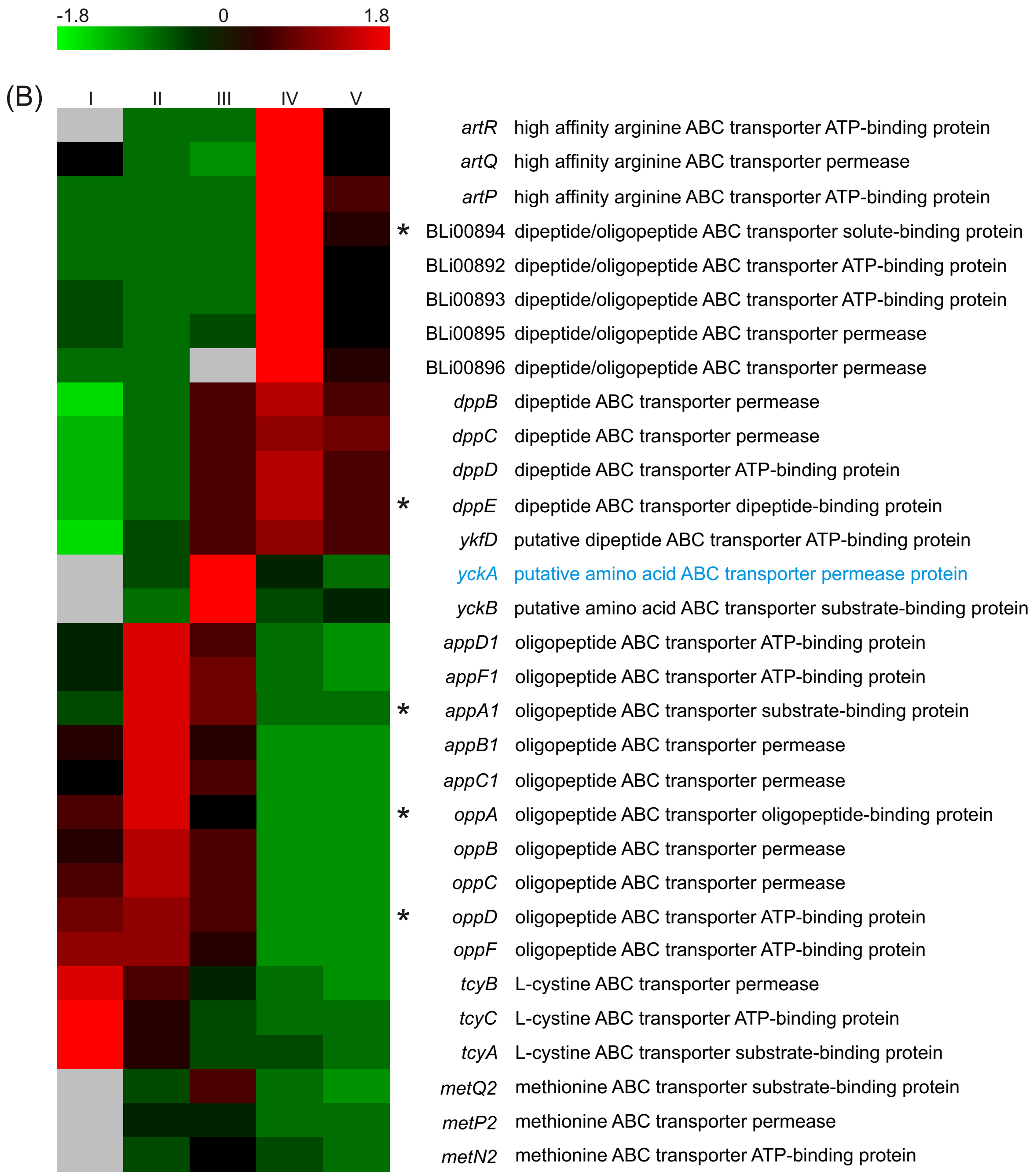

Figure S4 Amino acid transport. Heat map representation of Z-score transformed NPKM values. The depicted genes have been annotated as **(A)** amino acid or nitrogen transporters and **(B)** amino acid ABC transporter components. Please note that the figure does not give a complete list of genes involved in amino acid transport. Genes with an assigned antisense RNA [1] are marked in blue, asterisks indicate a detected protein spot for the respective gene (Additional File 2: Table S3) and statistically not significant values are marked by grey boxes.
Transporter genes with high transcript abundances during the early stages of the fermentation process are for example encoding a tryptophan transporter (trp), cystine (tcyABC) and methionine (metNPQ) ABC transporters and diverse proteins for uptake of alanine or unspecific amino acids. Additionally, the transcript of the ammonium transporter NrgA, especially required for ammonium transport at low ammonium concentrations in *B. subtilis* [2], is highly abundant at sampling point I. The only operon besides the dipeptide ABC transporters mentioned in the main text with distinct transcript abundance at the later sampling points encodes a high-affinity arginine ABC transporter (artPQR).

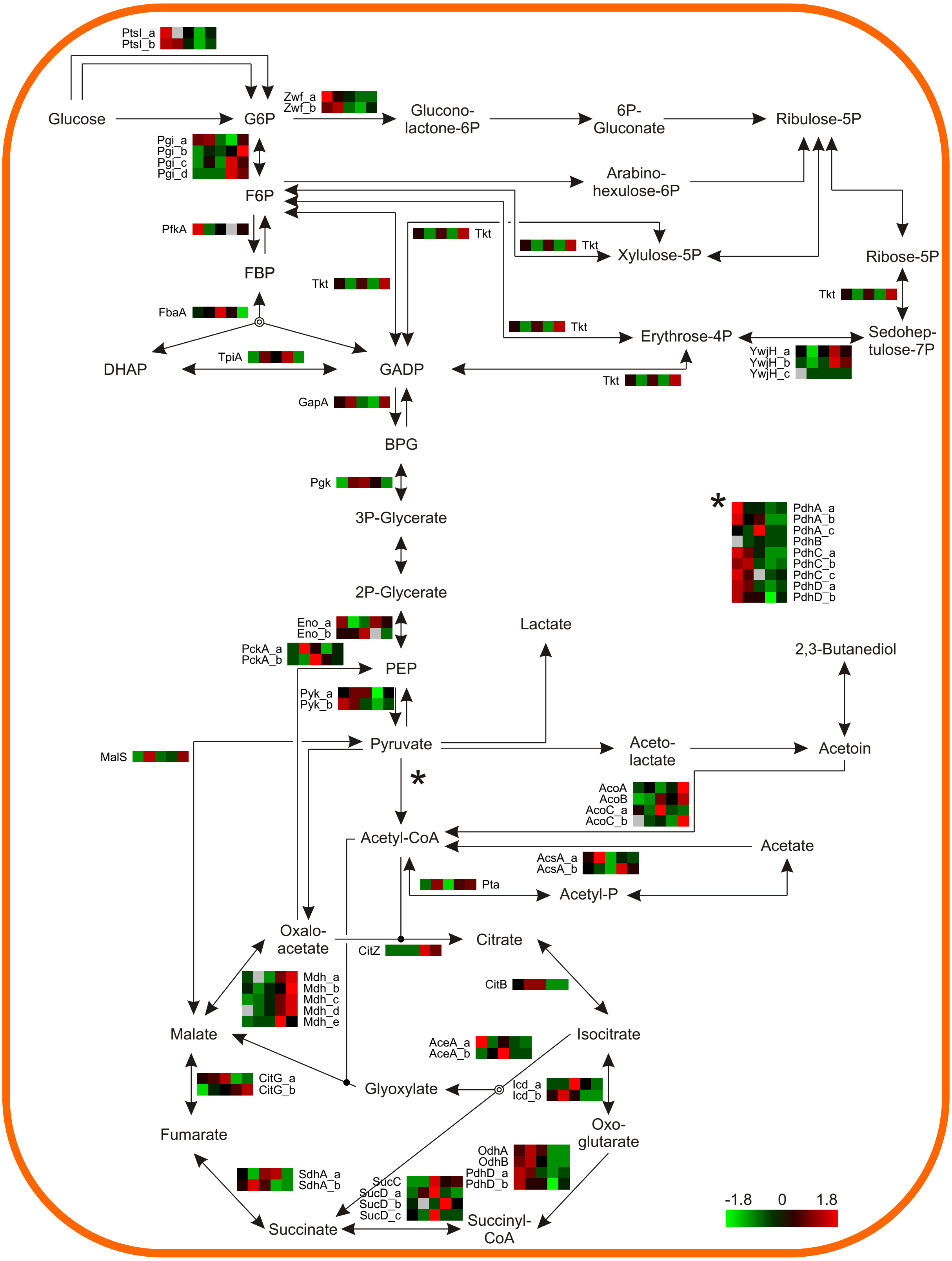

Figure S5 Proteome of the central carbon metabolism. Heat map representation of Z-score transformed protein spot volumes of proteins involved in central carbon metabolism. In cases where a specific protein is assigned to more than one spot, the particular spots are indicated by an ordering letter. Statistically not significant values are indicated by light grey boxes. For the corresponding transcriptome data please refer to Figure 7.

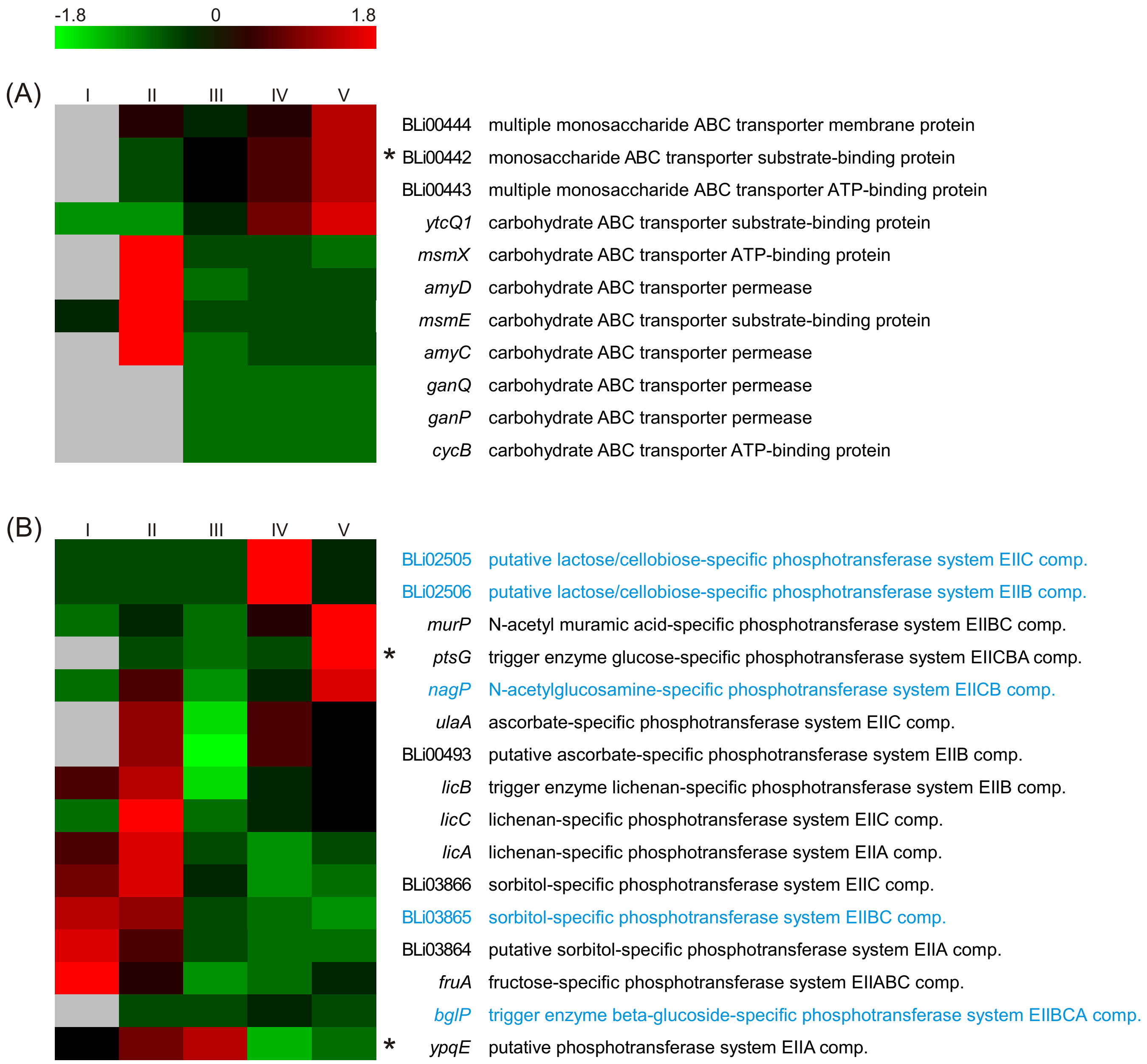

-1.8
0
1.8
(A)
I
II
III
IV
V
BLi00444 multiple monosaccharide ABC transporter membrane protein
* BLi00442 monosaccharide ABC transporter substrate-binding protein
BLi00443 multiple monosaccharide ABC transporter ATP-binding protein
ytcQ1 carbohydrate ABC transporter substrate-binding protein
msmX carbohydrate ABC transporter ATP-binding protein
amyD carbohydrate ABC transporter permease
msmE carbohydrate ABC transporter substrate-binding protein
amyC carbohydrate ABC transporter permease
ganQ carbohydrate ABC transporter permease
ganP carbohydrate ABC transporter permease
cycB carbohydrate ABC transporter ATP-binding protein
(B)
I
II
III
IV
V
BLi02505 putative lactose/cellobiose-specific phosphotransferase system EIIC comp.
BLi02506 putative lactose/cellobiose-specific phosphotransferase system EIIB comp.
murP N-acetyl muramic acid-specific phosphotransferase system EIIBC comp.
* ptsG trigger enzyme glucose-specific phosphotransferase system EIICBA comp.
nagP N-acetylglucosamine-specific phosphotransferase system EIICB comp.
ulaA ascorbate-specific phosphotransferase system EIIC comp.
BLi00493 putative ascorbate-specific phosphotransferase system EIIB comp.
licB trigger enzyme lichenan-specific phosphotransferase system EIIB comp.
licC lichenan-specific phosphotransferase system EIIC comp.
licA lichenan-specific phosphotransferase system EIIA comp.
BLi03866 sorbitol-specific phosphotransferase system EIIC comp.
BLi03865 sorbitol-specific phosphotransferase system EIIBC comp.
BLi03864 putative sorbitol-specific phosphotransferase system EIIA comp.
fruA fructose-specific phosphotransferase system EIIABC comp.
bglP trigger enzyme beta-glucoside-specific phosphotransferase system EIIBCA comp.
* ypqE putative phosphotransferase system EIIA comp.

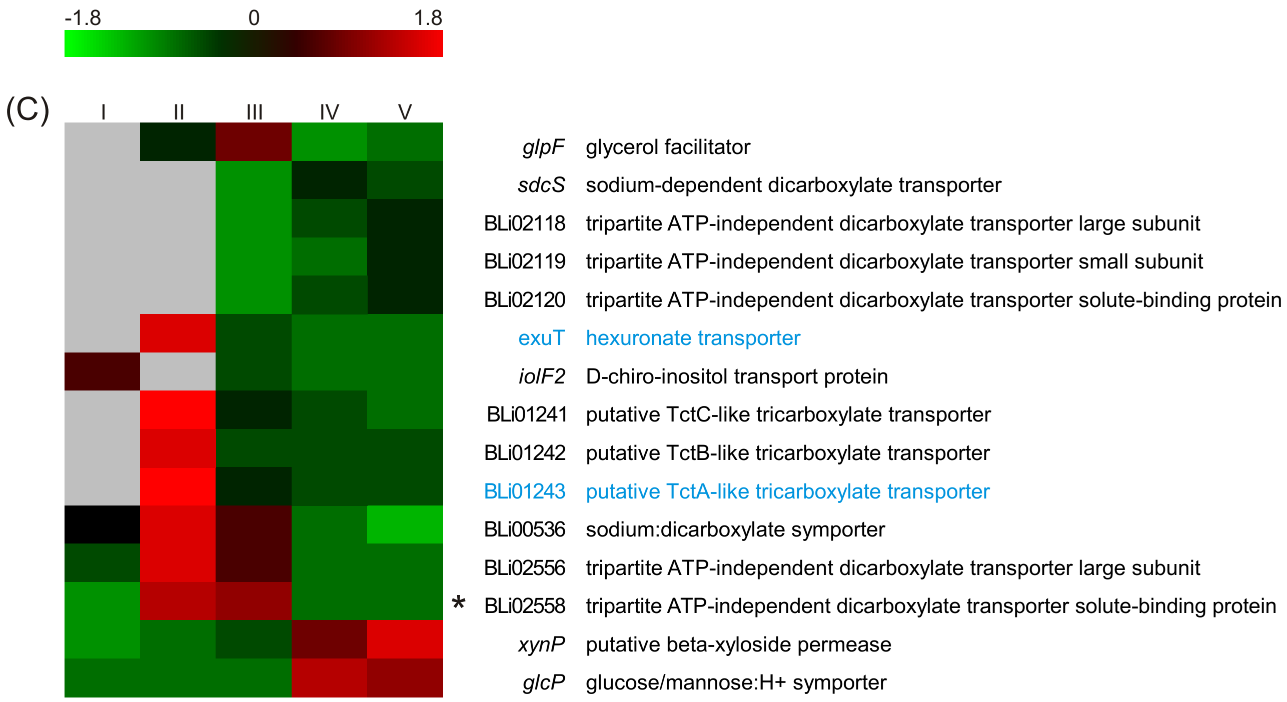

Figure S6 Carbohydrate transport. Heat map representation of Z-score transformed NPKM values. The depicted genes have been annotated as **(A)** carbohydrate ABC transporter components, **(B)** phosphotransferase system EII components and **(C)** further carbohydrate transporters. Please note that the figure does not give a complete list of genes involved in carbohydrate transport. Genes with an assigned antisense RNA [1] are marked in blue, asterisks indicate a detected protein spot for the respective gene (Additional File 2: Table S3) and statistically not significant values are marked by grey boxes.

At sampling point II, the transcript abundances indicate the consumption of the previously synthesized acetate (Figure 7). These results are supported by RNA abundance shifts of associated transporters. The transcript of a tripartite ATP-independent periplasmic dicarboxylate transporter (TRAP) of the TAXI type (BLi02556 and BLi02558), which is suggested as capable of acetate transport [3], is highly abundant.

During the late production stages, transcripts of transporters of diverse sugars (GlcP, XynP, BglP, SdcS) and cell wall components (MurP, NagP) are abundant. It is likely that this reaction can be accounted to the availability of such compounds due to cell lysis, as for example described at the onset of sporulation in *B. subtilis* [4], or shear effects in the fermenter.

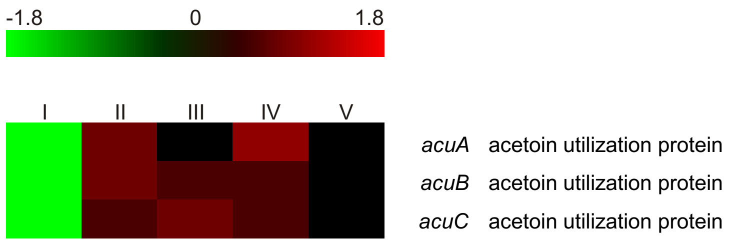

Figure S7 Acetoin utilization operon *acuABC*. Heat map representation of Z-score transformed NPKM values. The depicted genes are annotated as acetoin utilization operon *acuABC*.

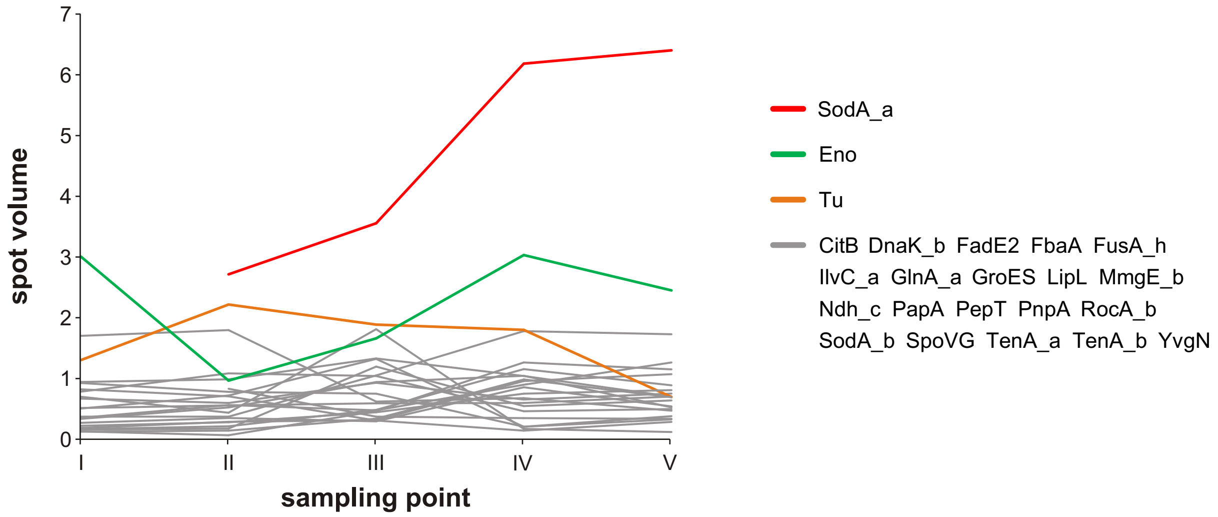

Figure S8 Most abundant proteins. Mean spot volumes of the most abundant proteins are plotted against sampling points (see also Additional File 2: Table S3). Grey lines represent protein spot volumes either higher than 0.8% at one sampling point or higher than 0.5% at all sampling points. Lines colored green, red and orange indicate the three most abundant protein spots Eno, Tu and SodA_a. In case a specific protein can be assigned to more than one spot, the particular spot is indicated by an ordering letter. Please note that statistically not significant values are not shown.

Highly abundant transcripts (Figure 2) for which predominant proteins could also be observed are coding for SodA, Tu and SpoVG. Other strongly synthesized proteins are, for example, the enolase Eno and proteins involved in carbon and amino acid metabolism or cofactor synthesis. They also comprise peptidases, the heat shock proteins GroES and DnaK and elongation factor G. Protein spots corresponding to the strongly transcribed genes *lanA1* and *lanA2* could not be identified. This was expected, as these proteins are probably exported by ABC transporters [5] and thus cannot be detected by proteome analysis of cytoplasmic proteins. The findings of highly abundant proteins match the results for *B. licheniformis* shown by Voigt et al. [6]. The only major exception is the absence of the flagellin protein Hag, which is also not highly abundant on transcript level. This effect is due to repression of *hag* gene expression in the presence of amino acids [7].

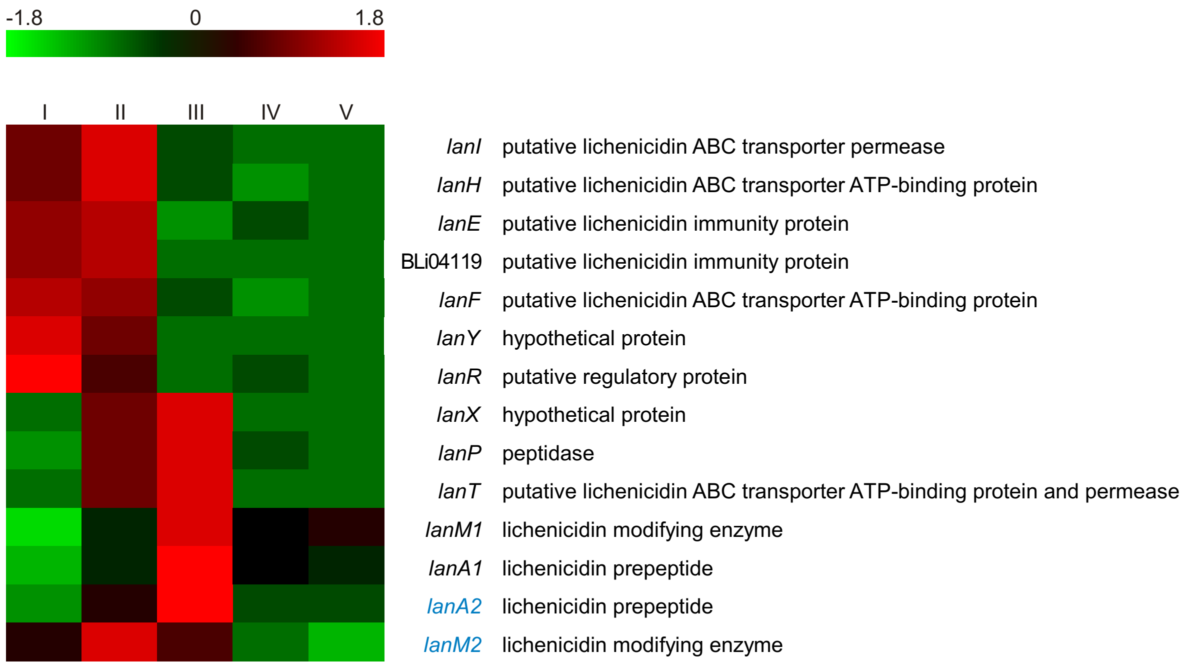

Figure S9 Lichenicidin gene cluster. Heat map representation of Z-score transformed NPKM values. The depicted genes have been identified as two-peptide lantibiotic lichenicidin-processing gene cluster Lan in *B. licheniformis* [5, 8]. Genes with an assigned antisense RNA [1] are marked in blue.

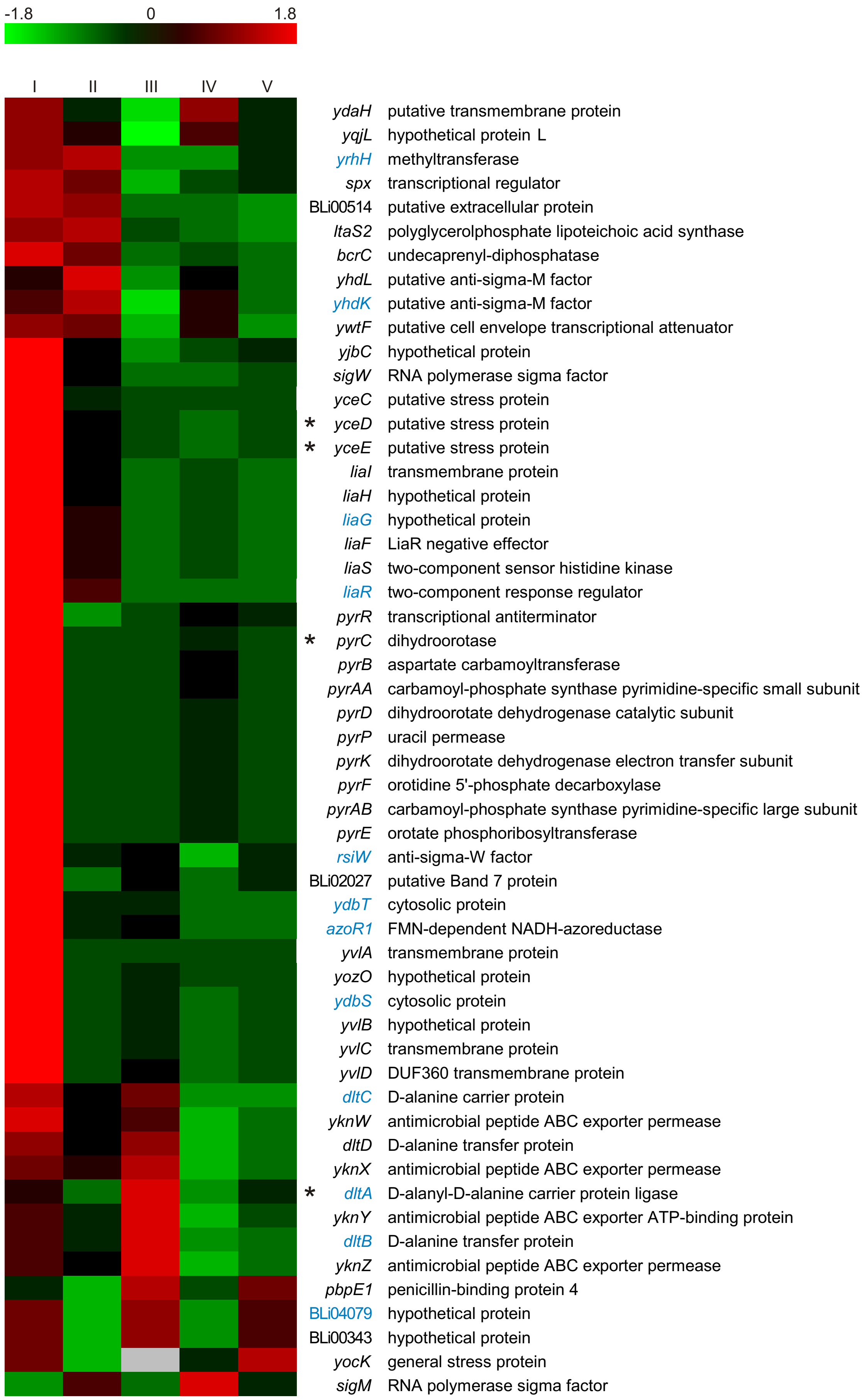
-1.8
0
1.8
I
II
III
IV
V
ydaH putative transmembrane protein
yqjL hypothetical protein L
yrhH methyltransferase
spx transcriptional regulator
BLi00514 putative extracellular protein
ltaS2 polyglycerolphosphate lipoteichoic acid synthase
bcrC undecaprenyl-diphosphatase
yhdL putative anti-sigma-M factor
yhdK putative anti-sigma-M factor
ywtF putative cell envelope transcriptional attenuator
yjbC hypothetical protein
sigW RNA polymerase sigma factor
yceC putative stress protein
* yceD putative stress protein
* yceE putative stress protein
liaI transmembrane protein
liaH hypothetical protein
liaG hypothetical protein
liaF LiaR negative effector
liaS two-component sensor histidine kinase
liaR two-component response regulator
pyrR transcriptional antiterminator
* pyrC dihydroorotase
pyrB aspartate carbamoyltransferase
pyrAA carbamoyl-phosphate synthase pyrimidine-specific small subunit
pyrD dihydroorotate dehydrogenase catalytic subunit
pyrP uracil permease
pyrK dihydroorotate dehydrogenase electron transfer subunit
pyrF orotidine 5'-phosphate decarboxylase
pyrAB carbamoyl-phosphate synthase pyrimidine-specific large subunit
pyrE orotate phosphoribosyltransferase
rsiW anti-sigma-W factor
BLi02027 putative Band 7 protein
ydbT cytosolic protein
azoR1 FMN-dependent NADH-azoreductase
yvlA transmembrane protein
yozO hypothetical protein
ydbS cytosolic protein
yvlB hypothetical protein
yvlC transmembrane protein
yvlD DUF360 transmembrane protein
dltC D-alanine carrier protein
yknW antimicrobial peptide ABC exporter permease
dltD D-alanine transfer protein
yknX antimicrobial peptide ABC exporter permease
* dltA D-alanyl-D-alanine carrier protein ligase
yknY antimicrobial peptide ABC exporter ATP-binding protein
dltB D-alanine transfer protein
yknZ antimicrobial peptide ABC exporter permease
pbpE1 penicillin-binding protein 4
BLi04079 hypothetical protein
BLi00343 hypothetical protein
yocK general stress protein
sigM RNA polymerase sigma factor

Figure S10 Cell envelope stress response. Heat map representation of Z-score transformed NPKM values. The depicted genes have been identified as marker genes for the *B. licheniformis* cell envelope stress response by Wecke et al. [9]. Genes with an assigned antisense RNA [1] are marked in blue, asterisks indicate a detected protein spot for the respective gene (Additional File 2: Table S3) and statistically not significant values are marked by grey boxes. The genes *yvnB*, *sigY*, *pbpX* and *yxlCDEFG* are not shown due to lacking transcript abundances (NPKM values <10).

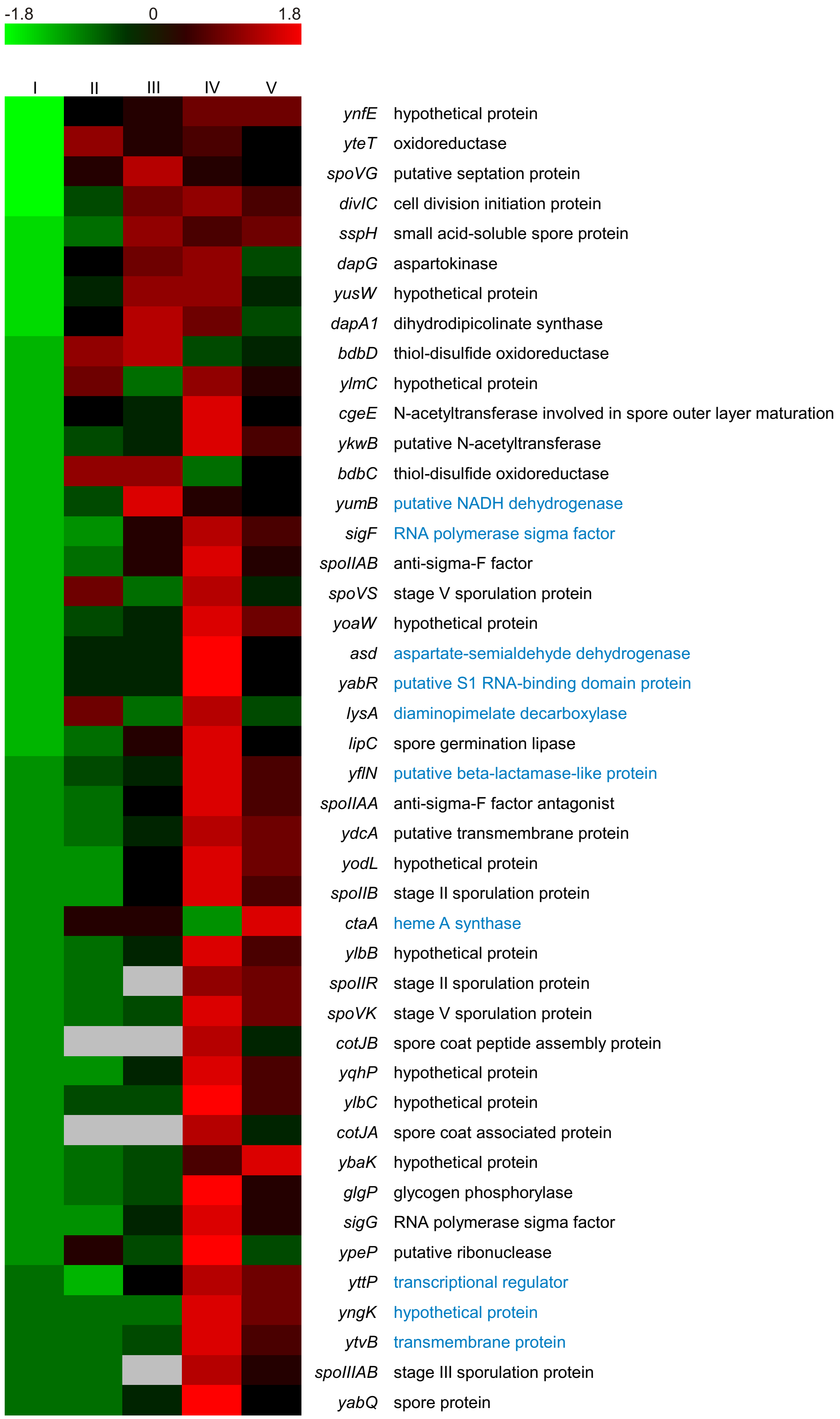
-1.8
0
1.8
I
II
III
IV
V
ynfE hypothetical protein
yteT oxidoreductase
spoVG putative septation protein
divIC cell division initiation protein
sspH small acid-soluble spore protein
dapG aspartokinase
yusW hypothetical protein
dapA1 dihydrodipicolinate synthase
bdbD thiol-disulfide oxidoreductase
ylmC hypothetical protein
cgeE N-acetyltransferase involved in spore outer layer maturation
ykwB putative N-acetyltransferase
bdbC thiol-disulfide oxidoreductase
yumB putative NADH dehydrogenase
sigF RNA polymerase sigma factor
spoIIAB anti-sigma-F factor
spoVS stage V sporulation protein
yoaW hypothetical protein
asd aspartate-semialdehyde dehydrogenase
yabR putative S1 RNA-binding domain protein
lysA diaminopimelate decarboxylase
lipC spore germination lipase
yflN putative beta-lactamase-like protein
spoIIAA anti-sigma-F factor antagonist
ydcA putative transmembrane protein
yodL hypothetical protein
spoIIB stage II sporulation protein
ctaA heme A synthase
ylbB hypothetical protein
spoIIR stage II sporulation protein
spoVK stage V sporulation protein
cotJB spore coat peptide assembly protein
yqhP hypothetical protein
ylbC hypothetical protein
cotJA spore coat associated protein
ybaK hypothetical protein
glgP glycogen phosphorylase
sigG RNA polymerase sigma factor
ypeP putative ribonuclease
yttP transcriptional regulator
yngK hypothetical protein
ytvB transmembrane protein
spoIIIAB stage III sporulation protein
yabQ spore protein

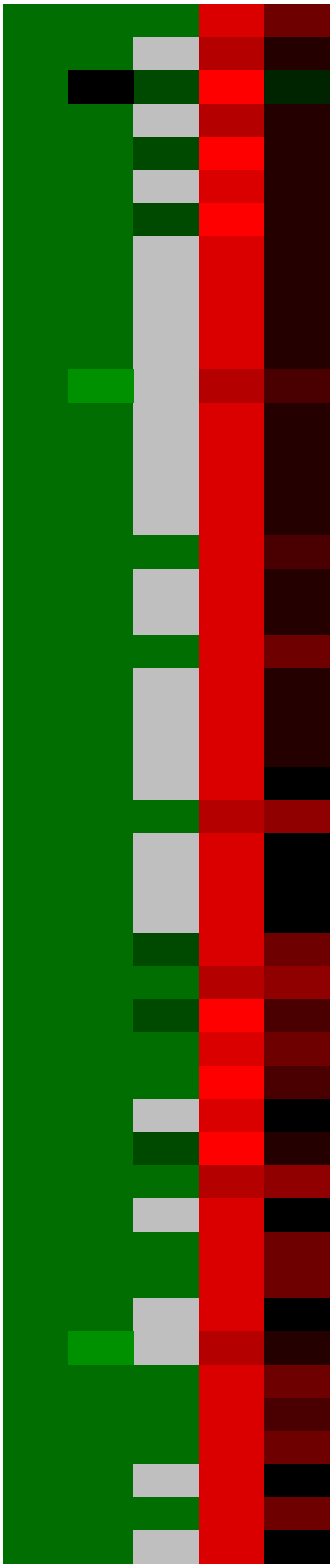
yhdB hypothetical protein
spoIIID stage III sporulation protein
ypfB hypothetical protein
spoIVA stage IV sporulation protein
spoVD stage V sporulation protein
yyaC sporulation protein
yqfC sporulation protein
spoIIIAG stage III sporulation protein
yabT putative serine/threonine-protein kinase
yqfD SigE-dependent sporulation gene
yabS hypothetical protein
yozD hypothetical protein
yqxA hypothetical protein
yngHB biotin/lipoyl attachment protein
yhxC putative oxidoreductase
yaaH glycoside hydrolase family protein
yhaL hypothetical protein
spoIIIAH stage III sporulation protein
yodQ 4-acetamidobutyryl-CoA deacetylase
yuzC sporulation protein
ydcC putative sporulation protein
yngE methylcrotonoyl-CoA carboxylase
ysxE positive modulator
sqhC squalene--hopene cyclase
ymfJ hypothetical protein
spoIIP cell wall hydrolase
prkA serine protein kinase
spoIIGA sigma-E factor-processing peptidase
yqhO putative acyl hydrolase/lysophospholipase
ytfI DUF2953 family protein
spoIISA stage II sporulation protein
spoVAC stage V sporulation protein
yhaX hypothetical protein
spoIIIAF stage III sporulation protein
glgA glycogen synthase
BLi03524 putative small acid-soluble spore protein
kamA L-lysine 2,3-aminomutase
yhcN forespore-specific protein
yhcV CBS domain protein
yngG 3-hydroxy-3-methylglutaryl-coenzyme A lyase
yteV sporulation protein
ykzD hypothetical protein
yjbA hypothetical protein
BLi01360 hypothetical protein
spoIIIAC stage III sporulation protein
sspA small acid-soluble spore protein
yngJ putative acyl-CoA dehydrogenase

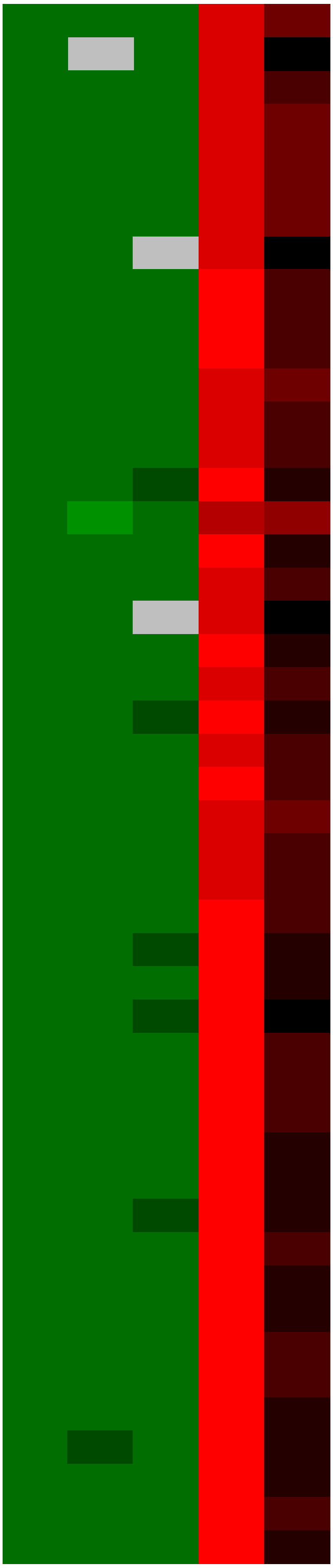

sspO	small acid-soluble spore protein
katX	catalase
sspI	small acid-soluble spore protein
BLi02230	hypothetical protein
sspP	small acid-soluble spore protein
BLi01951	hypothetical protein
yisY	AB hydrolase superfamily protein
yjbE	membrane protein
yngF	short chain enoyl-CoA hydratase
glgD	glycogen biosynthesis protein
spoIIQ	stage II sporulation protein
spoIVB	peptidase S
yqhR	hypothetical protein
yuzA	general stress protein
rsfA	putative transcriptional regulator
yqgO	hypothetical protein
yheD	hypothetical protein
yhcQ	hypothetical protein
yhbH	sporulation protein
yndM	hypothetical protein
yqhG	hypothetical protein
ywcB	DUF485 transmembrane protein
yrrD	forespore-specific sporulation protein
alr2	alanine racemase
azoR2	FMN-dependent NADH-azoreductase
cotO	spore coat morphogenetic protein
sspB	small acid-soluble spore protein
yqhV	hypothetical protein
sodF	putative superoxide dismutase
gerR	putative transcriptional regulator
yhfN	putative metalloprotease
yjaV	hypothetical protein
BLi00840	hypothetical protein
yhfW	putative Rieske 2Fe-2S iron-sulfur protein
dacF	D-alanyl-D-alanine carboxypeptidase
yrzE	transmembrane protein
accC1	acetyl-CoA carboxylase biotin carboxylase subunit
cwlC	N-acetylmuramoyl-L-alanine amidase
spoIIIAD	stage III sporulation protein
yodR	6-acetamido-3-oxohexanoate:acetyl-CoA CoA transferase beta subunit
glgB	1,4-alpha-glucan-branching enzyme
bofC	general stress protein
cotJC	spore coat peptide assembly protein
ywcA	Na+/solute symporter
ytlD	putative anion ABC transporter permease
lonB	Lon-like ATP-dependent protease
spoVAF1	stage V sporulation protein

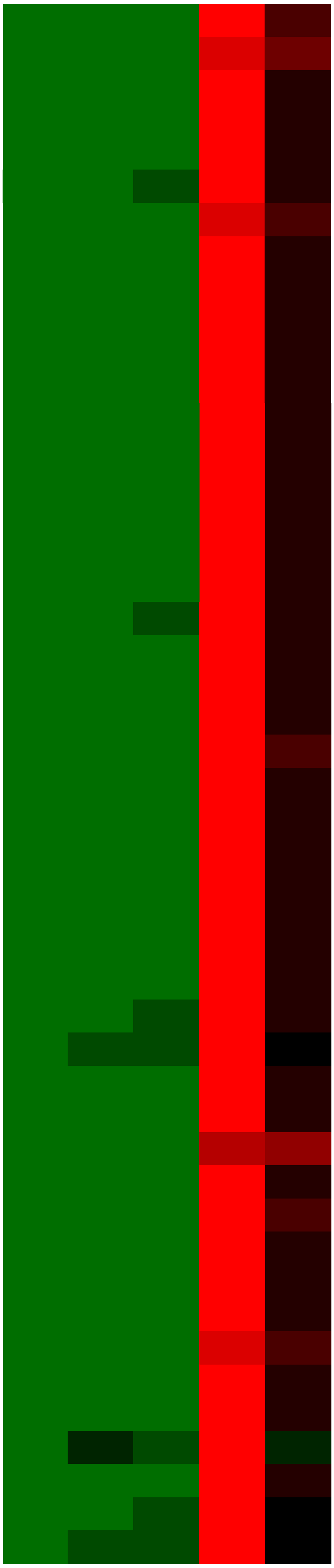

yurZ	putative alkylhydroperoxidase
yoaR	hypothetical protein
spoIVFB	stage IV sporulation protein
sspK	small acid-soluble spore protein
ytrH	sporulation membrane protein
ytrI	sporulation membrane protein
dacB	D-alanyl-D-alanine carboxypeptidase
yutH	spore coat protein
ycgF	putative amino acid efflux protein
spoIIIAE	stage III sporulation protein
spoIIIAA	stage III sporulation protein
ylaJ	hypothetical protein
spoIVFA	stage IV sporulation protein
yusN	putative spore coat protein
spmB	spore maturation protein
yodT	N(6)-acetyl-beta-lysine transaminase precursor
yodP	beta-lysine acetyltransferase
ydhD	putative sporulation-specific glycosylase
pdaB	polysaccharide deacetylase
ytlC	putative anion ABC transporter ATP-binding protein
ylzJ	hypothetical protein
yheC	hypothetical protein
yuiC	sporulation protein
ytlA	putative anion ABC transporter substrate-binding protein
spoVAA	stage V sporulation protein
yodS	6-acetamido-3-oxohexanoate:acetyl-CoA CoA transferase alpha subunit
yknT	putative sporulation protein
asnO	asparagine synthase
cotF	spore coat protein
yqfT	hypothetical protein
glgC	glucose-1-phosphate adenylyltransferase
gin	forespore-specific protein
spoIIM	stage II sporulation protein
pbpG	penicillin-binding protein 2D
sspF	small acid-soluble spore protein
yraD	spore coat protein F-like protein
yteA	sporulation protein
yjcA	sporulation protein
tgl	protein-glutamine gamma-glutamyltransferase
ydfS	UPF0702 transmembrane protein
seaA	spore envelope assembly protein
yokU	hypothetical protein
yitG	putative major facilitator superfamily protein
yqhQ	hypothetical protein
yitD	phosphosulfolactate synthase
ylbJ	putative sporulation integral membrane protein
ytvI	sporulation integral membrane protein

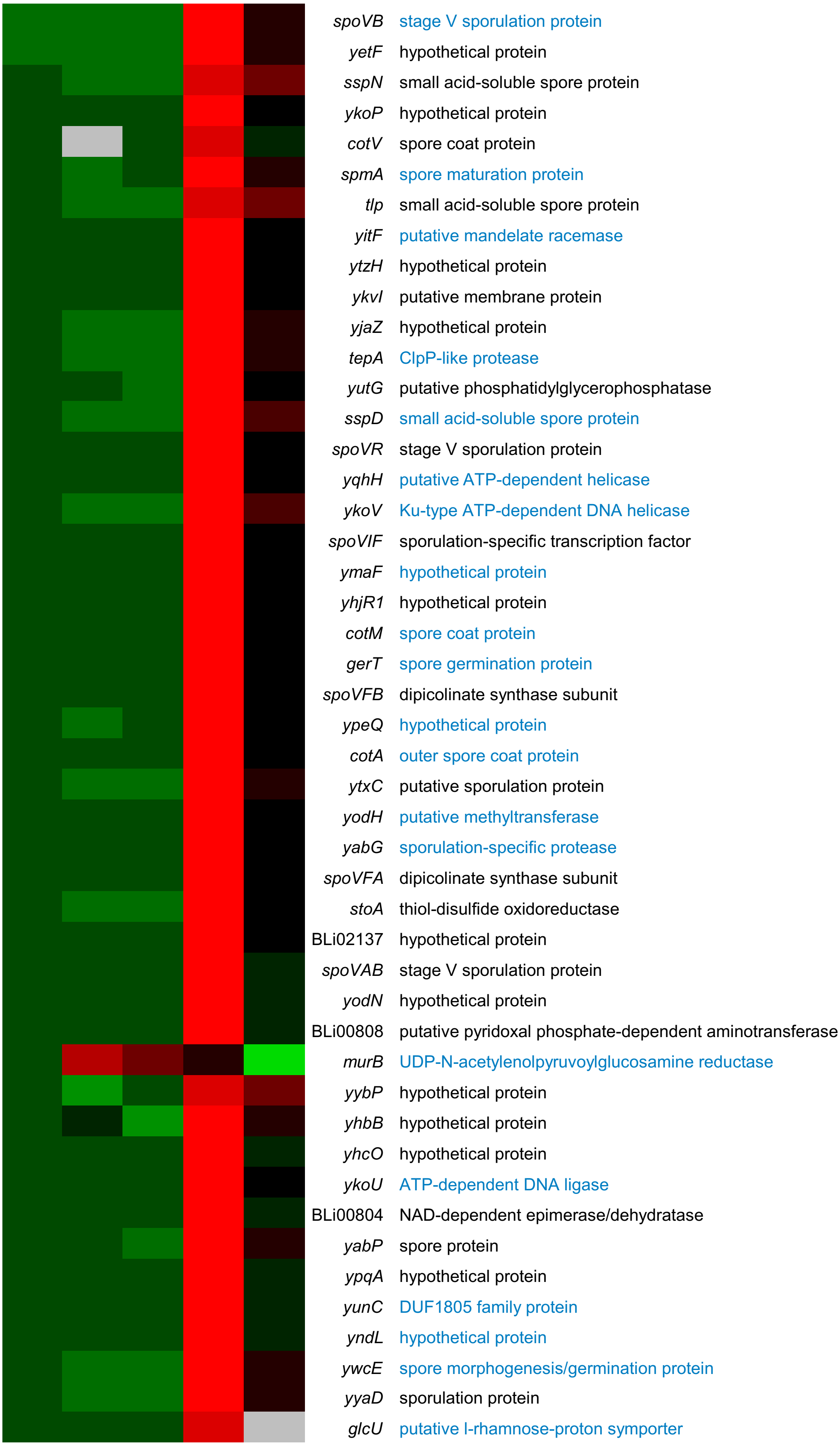
spoVB stage V sporulation protein
yetF hypothetical protein
sspN small acid-soluble spore protein
ykoP hypothetical protein
cotV spore coat protein
spmA spore maturation protein
tlp small acid-soluble spore protein
yitF putative mandelate racemase
ytzH hypothetical protein
ykvI putative membrane protein
yjaZ hypothetical protein
tepA ClpP-like protease
yutG putative phosphatidylglycerophosphatase
sspD small acid-soluble spore protein
spoVR stage V sporulation protein
yqhH putative ATP-dependent helicase
ykoV Ku-type ATP-dependent DNA helicase
spoVIF sporulation-specific transcription factor
ymaF hypothetical protein
yhjR1 hypothetical protein
cotM spore coat protein
gerT spore germination protein
spoVFB dipicolinate synthase subunit
ypeQ hypothetical protein
cotA outer spore coat protein
ytxC putative sporulation protein
yodH putative methyltransferase
yabG sporulation-specific protease
spoVFA dipicolinate synthase subunit
stoA thiol-disulfide oxidoreductase
BLi02137 hypothetical protein
spoVAB stage V sporulation protein
yodN hypothetical protein
BLi00808 putative pyridoxal phosphate-dependent aminotransferase
murB UDP-N-acetylenolpyruvoylglucosamine reductase
yybP hypothetical protein
yhbB hypothetical protein
yhcO hypothetical protein
ykoU ATP-dependent DNA ligase
BLi00804 NAD-dependent epimerase/dehydratase
yabP spore protein
ypqA hypothetical protein
yunC DUF1805 family protein
yndL hypothetical protein
ywcE spore morphogenesis/germination protein
yyaD sporulation protein
glcU putative l-rhamnose-proton symporter

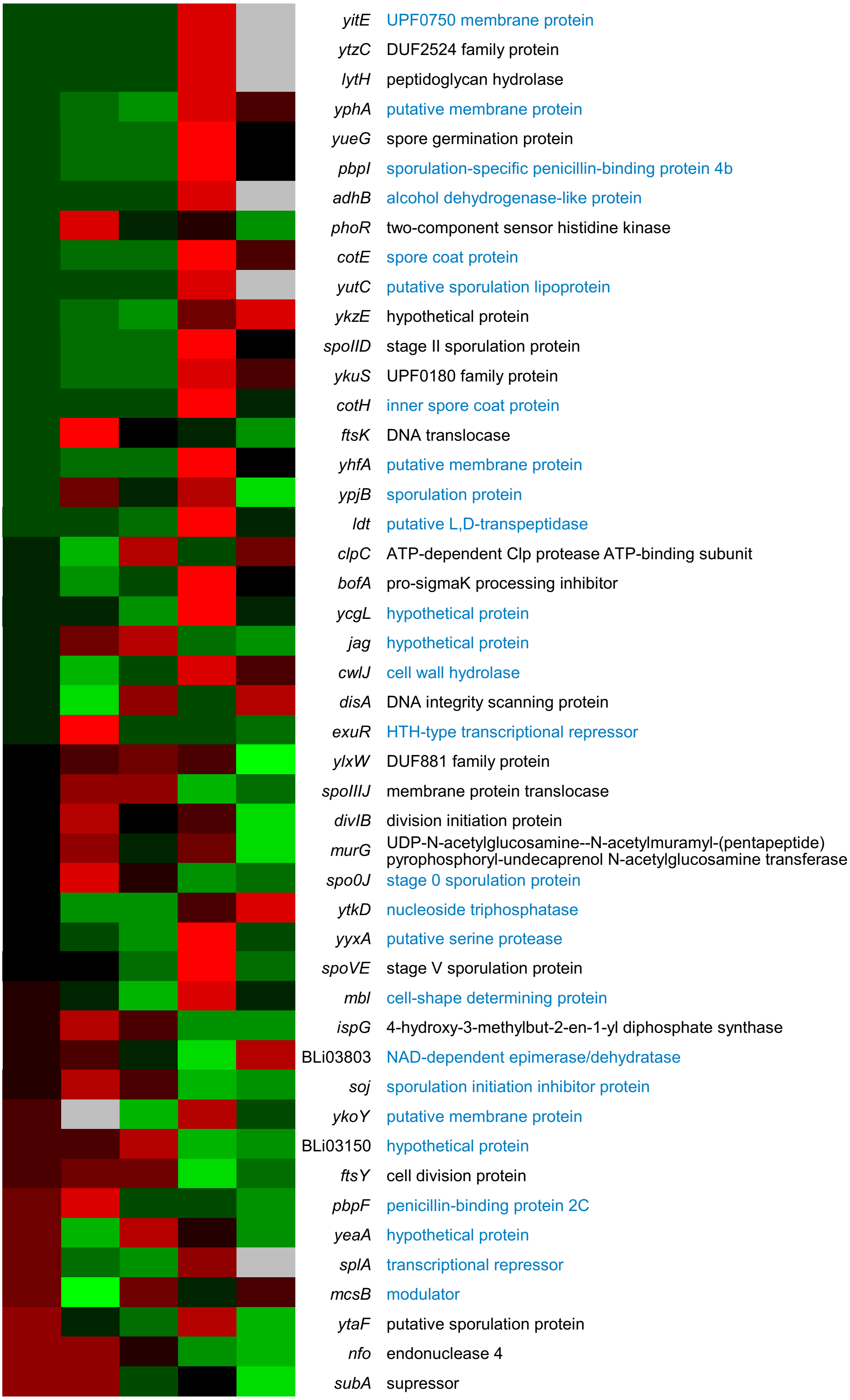

yitE UPF0750 membrane protein
ytzC DUF2524 family protein
lytH peptidoglycan hydrolase
yphA putative membrane protein
yueG spore germination protein
pbpI sporulation-specific penicillin-binding protein 4b
adhB alcohol dehydrogenase-like protein
phoR two-component sensor histidine kinase
cotE spore coat protein
yutC putative sporulation lipoprotein
ykzE hypothetical protein
spoIID stage II sporulation protein
ykuS UPF0180 family protein
cotH inner spore coat protein
ftsK DNA translocase
yhfA putative membrane protein
ypjB sporulation protein
ldt putative L,D-transpeptidase
clpC ATP-dependent Clp protease ATP-binding subunit
bofA pro-sigmaK processing inhibitor
ycgL hypothetical protein
jag hypothetical protein
cwlJ cell wall hydrolase
disA DNA integrity scanning protein
exuR HTH-type transcriptional repressor
ylxW DUF881 family protein
spoIIIJ membrane protein translocase
divIB division initiation protein
murG UDP-N-acetylglucosamine--N-acetylmuramyl-(pentapeptide) pyrophosphoryl-undecaprenol N-acetylglucosamine transferase
spo0J stage 0 sporulation protein
ytkD nucleoside triphosphatase
yyxA putative serine protease
spoVE stage V sporulation protein
mbl cell-shape determining protein
ispG 4-hydroxy-3-methylbut-2-en-1-yl diphosphate synthase
BLi03803 NAD-dependent epimerase/dehydratase
soj sporulation initiation inhibitor protein
ykoY putative membrane protein
BLi03150 hypothetical protein
ftsY cell division protein
pbpF penicillin-binding protein 2C
yeaA hypothetical protein
splA transcriptional repressor
mcsB modulator
ytaF putative sporulation protein
nfo endonuclease 4
subA supressor

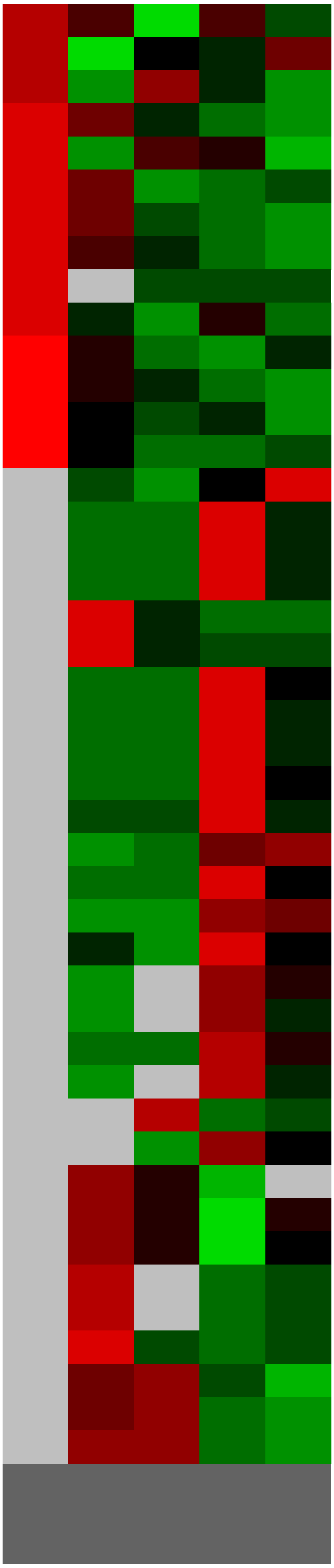

yrrS	DUF1510 family protein
mcsA	modulator
spoVT	stage V sporulation protein
ylxX	DUF881 family protein
yflJ	hypothetical protein
phoP2	two-component response regulator
sbp	small basic protein
fabL	NAD(P)-binding enoyl-(acyl-carrier-protein) reductase
yqfU	UPF0750 membrane protein
yozE	UPF0346 family protein
BLi04323	putative phage resolvase
rsmA	dimethyladenosine transferase
ytwI	DUF441 transmembrane protein
yrzA	hypothetical protein
ybbC	hypothetical protein
cotZ	spore coat protein
cotY	spore coat protein
cotX	spore coat protein
uxuA1	mannonate dehydratase
exuT	hexuronate transporter
splB	spore photoproduct lyase
ylbD	hypothetical protein
ylbE2	hypothetical protein
ymxH	hypothetical protein
yobW	sporulation membrane protein
ypzA	hypothetical protein
yqfZ	hypothetical protein
yqfX	hypothetical protein
yrzK	hypothetical protein
coxA	sporulation cortex protein
safA	hypothetical protein
ytfJ	sporulation protein
yunB	sporulation protein
uxaA	altronate hydrolase
ctpB	carboxy-terminal processing protease
tuaH	putative teichuronic acid biosynthesis glycosyltransferase
tuaG	putative teichuronic acid biosynthesis glycosyltransferase
tuaF	teichuronic acid biosynthesis protein
mmgC	butyryl-CoA dehydrogenase
mmgB	3-hydroxybutyryl-CoA dehydrogenase
mmgA	acetyl-CoA acetyltransferase
mmgD	2-methylcitrate synthase
mmgE	2-methylcitrate dehydratase
yqiQ	methylisocitrate lyase
BLi02833	hypothetical protein
BLi05032	hypothetical protein
uxaB	altronate oxidoreductase

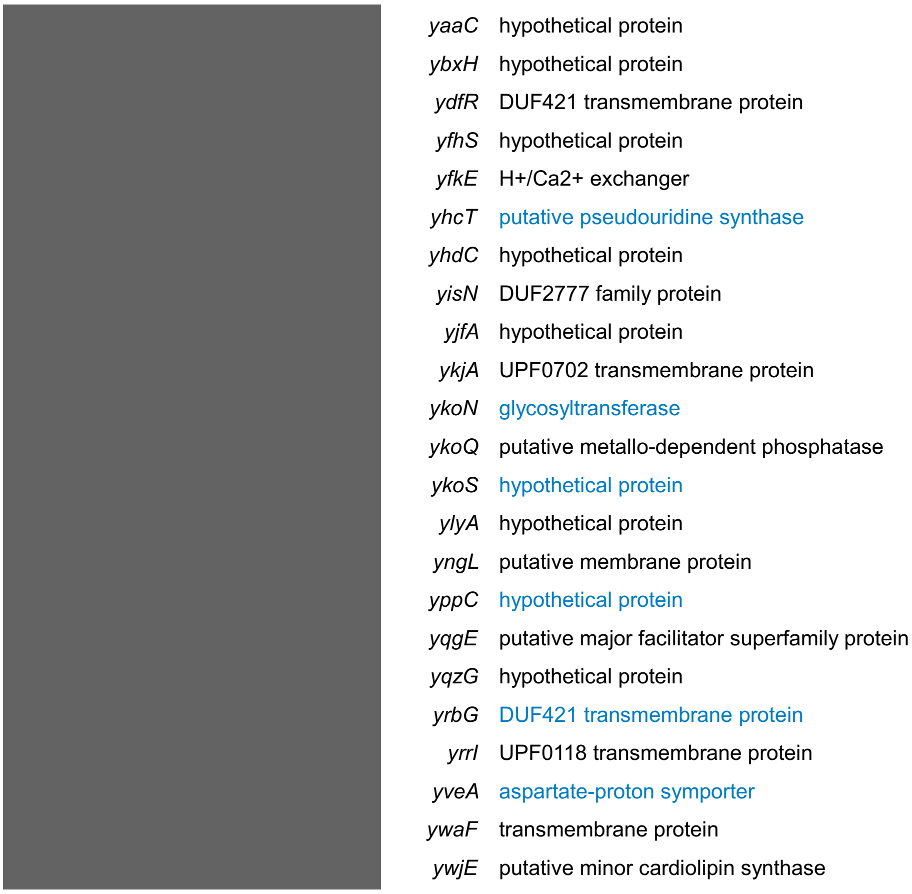

Figure S11 Sporulation. Heat map representation of Z-score transformed NPKM values. The depicted genes were identified as members of the sporulation cascade by reciprocal BLAST analysis [10] between *B. licheniformis* genes [1] and *B. subtilis* genes assigned to sporulation [11]. Genes with an assigned antisense RNA [1] are marked in blue, genes with NPKM values <10 at all sampling points are indicated by dark grey boxes, and statistically not significant values are indicated by light grey boxes.

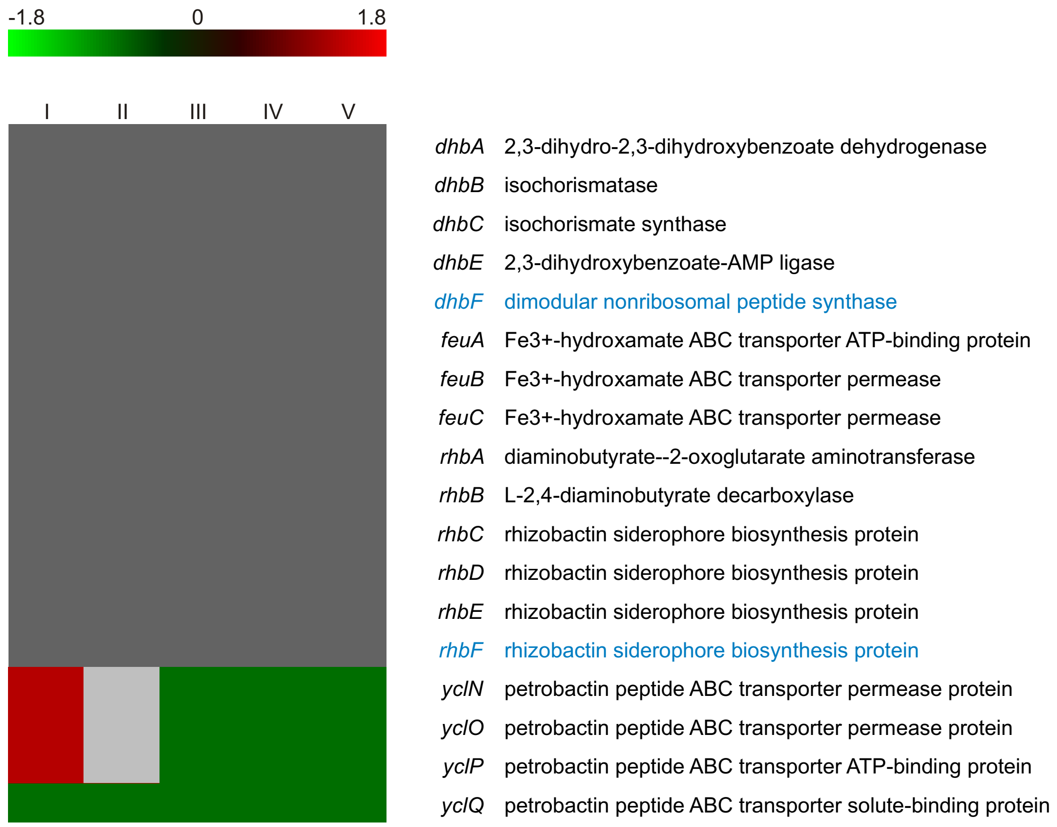

Figure S12 Iron starvation. Heat map representation of Z-score transformed NPKM values. The depicted genes have been identified as marker genes for *B. licheniformis* iron starvation by Nielsen et al. [12]. Genes with an assigned antisense RNA [1] are marked in blue, genes with NPKM values <20 at all sampling points are indicated by dark grey boxes and statistically not significant values are indicated by light grey boxes.

A well described indicator for iron starvation is the induction of siderophore anabolic genes (*dhbABCEF* and *rhbCDEF*) and siderophore importer genes (*feuABC* and *yclNOPQ*) [12], which are under control of the transcriptional regulator Fur [13]. With exception of the Ycl operon, these transcripts of these genes are not abundant during the fermentation process. This behavior indicates that iron starvation does not occur in any of the examined fermentation phases and corresponds to the observation that the Fur regulon is only induced during growth in minimal medium with restricted iron supply but not in rich medium [12, 14, 15], as used in this study.

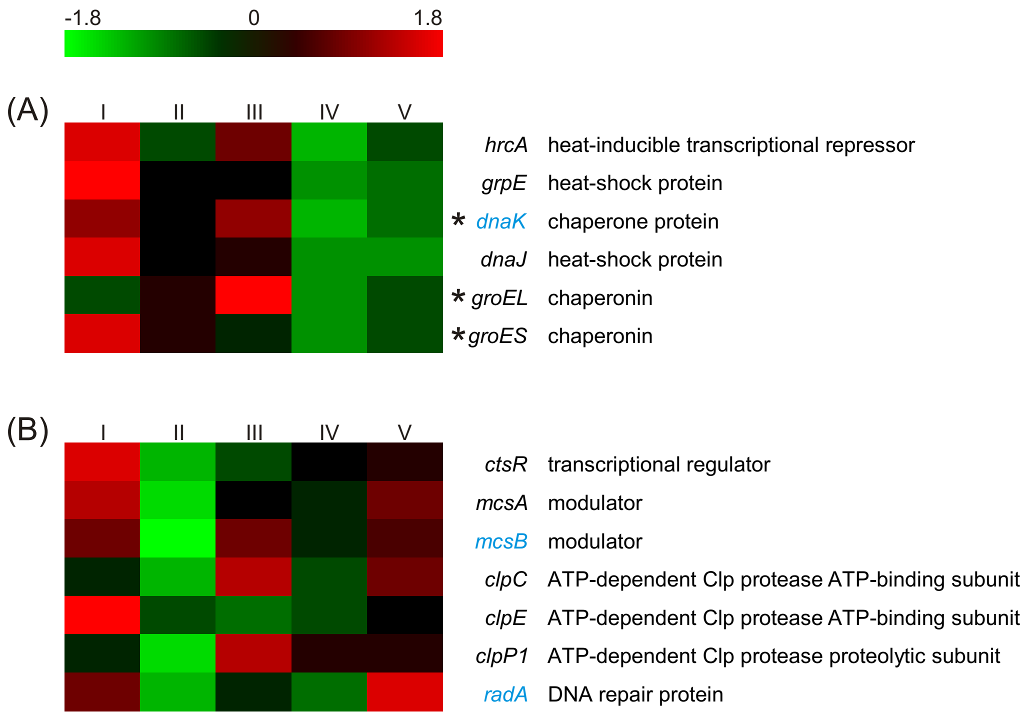

Figure S13 Heat shock response. Heat map representation of Z-score transformed NPKM values. The depicted **(A)** HrcA and **(B)** CtsR regulons have been identified as *B. licheniformis* heat stress markers by Nielsen et al. [12]. Genes with an assigned antisense RNA [1] are marked in blue, asterisks indicate a detected protein spot for the respective gene (Additional File 2: Table S3).

In cases of heat shock in *B. licheniformis*, the HrcA regulon including the *dnaK* and the *groE* operons and the CtsR regulon have been shown to be induced [12]. Furthermore, genes involved in iron and purine metabolism were upregulated, whereas the ABC transporter encoding *ytrABCEF* operon was repressed [12]. Since the here analyzed fermentation was not performed under heat shock conditions, the data did not show a typical heat shock response. Nevertheless, a transcriptional reaction to the fermentation temperature of 39 °C cannot be excluded, as the responses between moderate and severe heat shock can differ remarkably [16]. Despite the constant and moderate process temperature, differential expression of genes involved in a classic heat shock response [12, 17, 18] was observed. The ATP-dependent ClpCP protease shows changes in transcript abundance. However, the enzyme has been reported to be involved in proteolysis of misfolded proteins, which were caused by a variety of different stressors [19, 20] including for example the mentioned oxidative stress [14, 21, 22]. A reaction to non-heat stressors is also known for the chaperonins GroEL and GroES [23–25], which were transcribed with NPKM values >1000 at all sampling points or sampling point I, respectively. Although both chaperonins are encoded by the *groE* operon, the ratio of the transcript levels of the genes is highly variable between sampling points I and III – whereas *groES* declines over time, the *groEL* transcripts seem to be increasing. An explanation might be the processing of the bicistronic transcript into two monocistronic mRNAs. This effect has been described for *Agrobacterium tumefaciens* [26], in which the accumulation of *groEL* over time could also be shown. Furthermore, the determined GroEL protein amount (Additional File 2: Table S3) does not correlate with the amount of *groEL* mRNA, but shows the strongest abundance of protein at sampling point I.

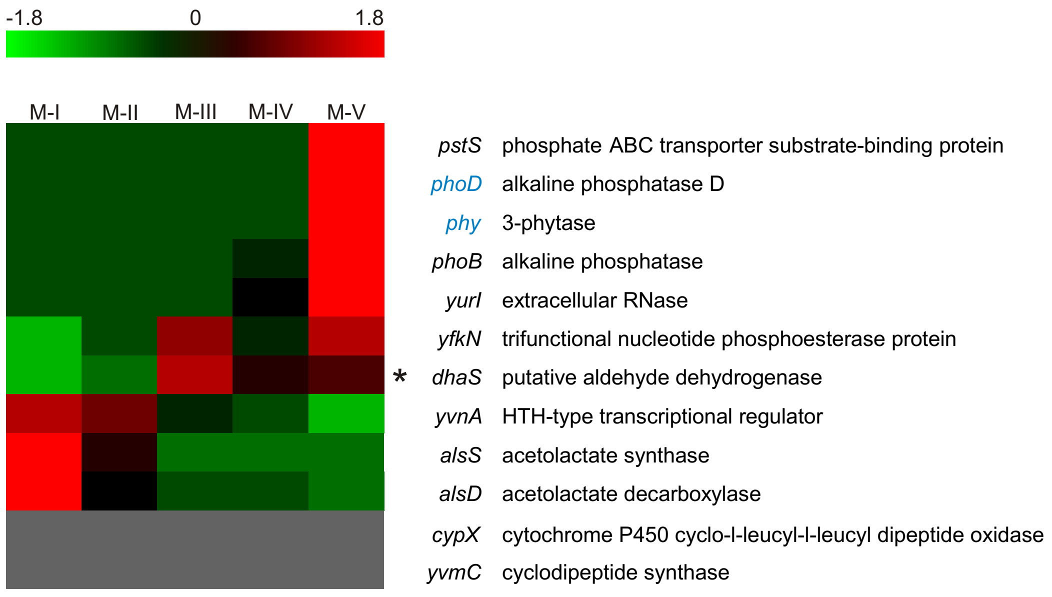

Figure S14 Phosphate starvation response. Heat map representation of Z-score transformed NPKM values. The depicted genes have been identified as marker genes for the *B. licheniformis* phosphate starvation response by Hoi et al. [27]. The figure shows the transcript abundances of those marker genes within the fermentation samples of one replicate (replicate M). Genes with an assigned antisense RNA [1] are marked in blue; asterisks indicate a detected protein spot for the respective gene Additional File 2: Table S3. Genes with NPKM values <10 at all sampling points are indicated by dark grey boxes.

At sampling point V, transcripts of *pstS* and the downstream *pst* operon encoding a phosphate ABC transporter (see also Figure 3), but also of *phy*, *phoB*, *phoD*, and *yfnK* are highly abundant. All of the mentioned genes are members of the Pho regulon, also known to provide a specific phosphate starvation stress response in *B. subtilis* [28, 29]. Furthermore, *yurI* and *dhaS* abundant as described by Hoi et al [27], supporting the inference of a phosphate shortage in this sample. However, no corresponding transcriptional response could be observed for the other fermentation samples (L and R) of sampling point V. This disparity coincides with an increased partial pressure of oxygen, starting ~1 h ahead of sampling point M-V (Additional File 1: Figure S1), which indicates a decreased metabolic activity in this sample at this fermentation stage. At the same time, the genes *yvnA*, *alsD,* and *alsS* show no increased transcript abundance. This is in accordance with the results obtained by Hoi et al. [27], who observed no induction of these genes in the early phases (~2 h) of declining metabolic activity after phosphate exhaustion. In addition, transcripts of genes involved in metabolic pathways, chemotaxis and especially teichoic acid synthesis were lacking, as also shown before [27]. The transcript abundance of the teichuronic acid synthesis operon (*tuaABCDEFG*), which enables the replacement of phosphate-rich teichoic acids, has been described as additional indicator of phosphate starvation in *B. subtilis* [28, 30] and could also be observed in sample M-V (data not shown). In summary, these results indicate that fermentation M is under phosphate limitation during the latest stage of the process. This finding might be a target for bioprocess optimization aiming at the prolongation of the fermentation process.

(A)

SRRXFLK

§§X##

(B)

MRKKSFWL

§§XX##

Figure S15 Putative Tat signal peptide of Subtilisin Carlsberg. (A) Tat signal peptides contain a conserved twin-arginine motif, defined as SRRXFLK, in which the consecutive arginine residues are almost always invariant [31]. Nevertheless, one arginine residue (§), but not both, can be replaced by a lysine residue [32, 33]. The other motif residues occur with a frequency of >50%, whereas the amino acids at the # positions are usually hydrophobic and the amino acid at the X position is polar [31, 34]. **(B)** The signal peptide of Subtilisin Carlsberg matches the Tat model motif with the exception of two hydrophilic residues in the middle positions, a condition not considered an exclusion criterion for a putative Tat signal peptide motif [35, 36]. In Sec signal peptides, helix breaking glycine or proline residues are often found in the middle of the hydrophobic domain following the N-terminal domain [37]. These are not found in *B. subtilis* Tat signal peptides [36] and also not in the signal peptide of Subtilisin Carlsberg.

References

1. Wiegand S, Dietrich S, Hertel R, Bongaerts J, Evers S, Volland S, Daniel R, Liesegang H: **RNA-Seq of *Bacillus licheniformis*: active regulatory RNA features expressed within a productive fermentation**. *BMC Genomics* 2013, revised.

2. Detsch C, Stülke J: **Ammonium utilization in *Bacillus subtilis*: transport and regulatory functions of NrgA and NrgB**. *Microbiology+* 2003, **149**:3289–3297.

3. Bakermans C, Sloup RE, Zarka DG, Tiedje JM, Thomashow MF: **Development and use of genetic system to identify genes required for efficient low-temperature growth of *Psychrobacter arcticus* 273-4.** *Extremophiles* 2009, **13**:21–30.

4. González-Pastor JE: **Cannibalism: a social behavior in sporulating *Bacillus subtilis*.** *FEMS Microbiol Rev* 2011, **35**:415–24.

5. Dischinger J, Josten M, Szekat C, Sahl H-G, Bierbaum G: **Production of the novel two-peptide lantibiotic lichenicidin by *Bacillus licheniformis* DSM 13.** *PLoS One* 2009, **4**:e6788.

6. Voigt B, Schweder T, Becher D, Ehrenreich A, Gottschalk G, Feesche J, Maurer K-H, Hecker M: **A proteomic view of cell physiology of *Bacillus licheniformis*.** *Proteomics* 2004, **4**:1465–90.

7. Bergara F, Ibarra C, Iwamasa J, Patarroyo JC, Aguilera R, Ma LM: **CodY Is a Nutritional Repressor of Flagellar Gene Expression in *Bacillus subtilis***. *J Bacteriol* 2003, **185**:3118–3126.

8. Begley M, Cotter PD, Hill C, Ross RP: **Identification of a novel two-peptide lantibiotic, lichenicidin, following rational genome mining for LanM proteins.** *Appl Environ Microbiol* 2009, **75**:5451–60.

9. Wecke T, Veith B, Ehrenreich A, Mascher T: **Cell envelope stress response in *Bacillus licheniformis*: integrating comparative genomics, transcriptional profiling, and regulon mining to decipher a complex regulatory network.** *J Bacteriol* 2006, **188**:7500–11.

10. Lechner M, Findeiss S, Steiner L, Marz M, Stadler PF, Prohaska SJ: **Proteinortho: detection of (co-)orthologs in large-scale analysis.** *BMC Bioinformatics* 2011, **12**:124.

11. Mäder U, Schmeisky AG, Flórez LA, Stülke J: **SubtiWiki--a comprehensive community resource for the model organism *Bacillus subtilis*.** *Nucleic Acids Res* 2012, **40**:D1278–87.

12. Nielsen AK, Breüner A, Krzystanek M, Andersen JT, Poulsen TA, Olsen PB, Mijakovic I, Rasmussen MD: **Global transcriptional analysis of *Bacillus licheniformis* reveals an overlap between heat shock and iron limitation stimulon.** *J Mol Microbiol Biotechnol* 2010, **18**:162–73.

13. Ollinger J, Song K-B, Antelmann H, Hecker M, Helmann JD: **Role of the Fur Regulon in Iron Transport in *Bacillus subtilis***. *J Bacteriol* 2006, **188**:3664–3673.

14. Mostertz J, Scharf C, Hecker M, Homuth G: **Transcriptome and proteome analysis of *Bacillus subtilis* gene expression in response to superoxide and peroxide stress**. *Microbiology+* 2004, **150**:497–512.

15. Helmann JD, Wu MFW, Gaballa A, Kobel PA, Morshedi MM, Fawcett P, Paddon C: **The Global Transcriptional Response of *Bacillus subtilis* to Peroxide Stress Is Coordinated by Three Transcription Factors**. *J Bacteriol* 2003, **185**:243.

16. Barreiro C, Nakunst D, Hüser AT, De Paz HD, Kalinowski J, Martín JF: **Microarray studies reveal a "differential response" to moderate or severe heat shock of the HrcA- and HspR-dependent systems in *Corynebacterium glutamicum*.** *Microbiology+* 2009, **155**:359–72.

17. Helmann JD, Wu MFW, Kobel PA, Gamo F-J, Wilson M, Morshedi MM, Navre M, Paddon C: **Global Transcriptional Response of *Bacillus subtilis* to Heat Shock** *J Bacteriol* 2001, **183**:7318.

18. Schumann W: **The *Bacillus subtilis* heat shock stimulon.** *Cell Stress Chaperon* 2003, **8**:207–17.

19. Krüger E, Witt E, Ohlmeier S, Hanschke R, Hecker M: **The Clp proteases of*Bacillus subtilis* are directly involved in degradation of misfolded proteins.** *J Bacteriol* 2000, **182**:3259–65.

20. Frees D, Savijoki K, Varmanen P, Ingmer H: **Clp ATPases and ClpP proteolytic complexes regulate vital biological processes in low GC, Gram-positive bacteria.** *Mol Microbiol* 2007, **63**:1285–95.

21. Leichert LIO, Scharf C, Hecker M: **Global Characterization of Disulfide Stress in *Bacillus subtilis*.** *J Bacteriol* 2003, **185**:1967.

22. Schroeter R, Voigt B, Jürgen B, Methling K, Pöther D-C, Schäfer H, Albrecht D, Mostertz J, Mäder U, Evers S, Maurer K-H, Lalk M, Mascher T, Hecker M, Schweder T: **The peroxide stress response of *Bacillus licheniformis*.** *Proteomics* 2011, **11**:2851–66.

23. Babu MMG, Sridhar J, Gunasekaran P: **Global transcriptome analysis of *Bacillus cereus* ATCC 14579 in response to silver nitrate stress.** *J Nanobiotechnology* 2011, **9**:49.

24. Lee N, Yeo I, Park J, Hahm Y: **Growth inhibition and induction of stress protein, GroEL, of *Bacillus cereus* exposed to antibacterial peptide isolated from Bacillus subtilis SC-8**. *Appl Biochem Biotechnol* 2011, **165**:235–42.

25. Seydlová G, Halada P, Fišer R, Toman O, Ulrych A, Svobodová J: **DnaK and GroEL chaperones are recruited to the *Bacillus subtilis* membrane after short-term ethanol stress**. *J Appl Microbiol* 2012, **112**:765–74.

26. Segal G, Ron E: **The *groESL* Operon of *Agrobacterium tumefaciens*: Evidence for Heat Shock-Dependent mRNA cleavage**. *J Bacteriol* 1995, **177**:750–57.

27. Hoi LT, Voigt B, Jürgen B, Ehrenreich A, Gottschalk G, Evers S, Feesche J, Maurer K-H, Hecker M, Schweder T: **The phosphate-starvation response of *Bacillus licheniformis*.** *Proteomics* 2006, **6**:3582–601.

28. Allenby NEE, Connor NO, Prágai Z, Ward C, Wipat A , Harwood CR: **Genome-Wide Transcriptional Analysis of the Phosphate Starvation Stimulon of *Bacillus subtilis***. *J Bacteriol* 2005, **187**:8063.

29. Antelmann H, Scharf C, Hecker M: **Phosphate Starvation-Inducible Proteins of *Bacillus subtilis*: Proteomics and Transcriptional Analysis**. *J Bacteriol* 2000, **182**:4478.

30. Liu W, Hulett FM: **Comparison of PhoP binding to the *tuaA* promoter with PhoP binding to other Pho-regulon promoters establishes a *Bacillus subtilis* Pho core binding site**. *Microbiology+* 1998, **144**:1443–1450.

31. Palmer T, Berks BC: **The twin-arginine translocation (Tat) protein export pathway.** *Nat Rev Microbiol* 2012, **10**:483–96.

32. DeLisa MP, Samuelson P, Palmer T, Georgiou G: **Genetic analysis of the twin arginine translocator secretion pathway in bacteria.** *J Biol Chem* 2002, **277**:29825–31.

33. Ize B, Gerard F, Zhang M, Chanal A, Voulhoux R, Palmer T, Filloux A, Wu L -F: **In vivo Dissection of the Tat Translocation Pathway in *Escherichia coli***. *J Mol Biol* 2002, **317**:327–335.

34. Kouwen TRHM, van der Ploeg R, Antelmann H, Hecker M, Homuth G, Mäder U, van Dijl JM: **Overflow of a hyper-produced secretory protein from the *Bacillus* Sec pathway into the Tat pathway for protein secretion as revealed by proteogenomics.** *Proteomics* 2009, **9**:1018–32.

35. Berks BC: **A common export pathway for proteins binding complex redox cofactors?** *Mol Microbiol* 1996, **22**:393–404.

36. Van Dijl JM, Braun PG, Robinson C, Quax WJ, Antelmann H, Hecker M, Müller J, Tjalsma H, Bron S, Jongbloed JDH: **Functional genomic analysis of the *Bacillus subtilis* Tat pathway for protein secretion.** *J Biotechnol* 2002, **98**:243–54.

37. De Vrije GJ, Batenburg AM, Killian JA, De Kruijff B: **Lipid involvement in protein translocation in *Escherichia coli***. *Mol Microbiol* 1990, **4**:143–150.
